# Outer Membrane Vesicles Mediate the Secretion and Nuclear Trafficking of a Bacterial Nucleomodulin

**DOI:** 10.1002/jev2.70286

**Published:** 2026-04-30

**Authors:** Jack K. Emery, Variya Nemidkanam, Nina Colon, Kate R. J. Friesen, Lena H. M. Le, Angus R. Cramond, Maxine Yap, Mônica S. Barbosa, Georgie Wray‐McCann, David J. McGee, Natalia Castaño‐Rodríguez, Dongmei Tong, Caroline Skene, Laurent Terradot, Richard L. Ferrero

**Affiliations:** ^1^ Centre for Innate Immunity and Infectious Diseases Hudson Institute of Medical Research Melbourne Australia; ^2^ Department of Molecular and Translational Sciences Monash University Melbourne Australia; ^3^ Clinical Biochemistry and Molecular Medicine Department of Clinical Chemistry Faculty of Allied Health Sciences Chulalongkorn University Bangkok Thailand; ^4^ Biomedicine Discovery Institute Department of Microbiology Monash University Melbourne Australia; ^5^ Instituto de Patologia Tropical e Saúde Pública Federal University of Goiás Goiânia Brasil; ^6^ Department of Microbiology and Immunology Louisiana State University Health Sciences Center Shreveport Shreveport Louisiana USA; ^7^ School of Biotechnology and Biomolecular Science University of New South Wales Sydney Australia; ^8^ Laboratory of Molecular Microbiology and Structural Biochemistry Centre National de la Recherche Scientifique UMR5086, Université de Lyon Lyon France

**Keywords:** *Helicobacter pylori*, outer membrane vesicles, OMVs, extracellular vesicles, tumour necrosis factor‐α‐inducing protein, Tipα, nucleomodulin

## Abstract

Nucleomodulins are a family of bacterial virulence proteins that traffic to the nucleus of host cells to disrupt nuclear processes and, in some cases, promote carcinogenesis. The mechanisms by which nucleomodulins are secreted and injected into host cells are not entirely clear. We hypothesised that bacterial extracellular vesicles (EVs), also known as outer membrane vesicles, may represent a novel mechanism for nucleomodulin delivery to host cells. To address this, we studied the role of EVs in the secretion of *Helicobacter pylori* tumour necrosis factor‐α‐inducing protein (Tipα), a protein that was reported to undergo nuclear trafficking and to promote carcinogenesis. Importantly, we showed that most Tipα present in *H. pylori* culture supernatants is associated with EVs and, moreover, is encapsulated within these particles in its biologically active dimeric form. Confocal microscopy and cell fractionation studies demonstrated that EVs carry Tipα to the nuclear compartment of host cells and can bind host DNA. EVs from isogenic *tipA* mutants induced greater pro‐inflammatory cytokine responses in host cells than EVs from wild‐type bacteria; however, this effect was dependent on the EV isolation method. We propose that EVs represent a new mechanism by which bacteria secrete and deliver nucleomodulins to their target cells, resulting in altered immune responses and possibly carcinogenesis.

## Graphical abstract

(A) We propose that *Helicobacter pylori* secretes a nucleomodulin, Tumour Necrosis Factor‐α‐inducing protein (Tipα), predominantly via extracellular vesicles (EVs), also known as outer membrane vesicles, rather than as free protein. (B) EVs carrying Tipα enter eukaryotic cells by endocytosis and independently of surface‐expressed nucleolin, then traffic to the nuclear compartment. EVs and its Tipα cargo bind DNA to regulate inflammatory and immune gene expression, potentially leading to oncogenesis.

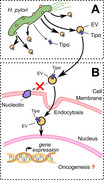

## 1 Introduction

Pathogenic bacteria employ many mechanisms to colonise and disrupt host cell processes. Secretion of virulence factors is one major mechanism bacteria use to cause disease in the host. One group of secreted virulence proteins, the ‘nucleomodulins’, traffic to the host cell nucleus and modulate nuclear processes, such as through epigenetic changes (Le et al. 2021). Nucleomodulins were first identified in phytopathogens (Hanford et al. 2021), with the best‐known example being *Agrobacterium tumefaciens* (Bierne and Pourpre 2020; Hanford et al. 2021). This bacterium encodes a tumour‐inducing (Ti) plasmid that is injected into plant cells via its type 4 secretion system (T4SS) and becomes integrated into the host genome (Bierne and Pourpre 2020; Hanford et al. 2021). Expression of Ti genes causes aberrant proliferation of the infected plant cells and the production of nutrients required for bacterial survival.

Several human bacterial pathogens have now been identified to also secrete nucleomodulins. These proteins were shown to upregulate inflammatory responses in host cells (Zhu et al. 2009; Lebreton et al. 2011), whereas others repressed nuclear factor‐κB (NF‐κB)‐dependent and innate immune genes via histone modifications (Arbibe et al. 2007; Rolando et al. 2013). Nucleomodulins have been shown to be secreted via known systems, including the general Sec secretion system (Bierne and Pourpre 2020; Hanford et al. 2021) or type‐1 (Zhu et al. 2009), ‐3 (Arbibe et al. 2007) or ‐4 secretion apparatuses (Rolando et al. 2013). In many cases, however, the secretion mechanisms for members of this protein family are not known (Hanford et al. 2021).

The causative agent of gastric cancer, *Helicobacter pylori*, secretes a novel protein, tumour necrosis factor‐α‐inducing protein (Tipα), which has characteristics of a nucleomodulin. *H. pylori* Tipα shares 94.3% homology to the membrane‐associated protein, HP‐MP1, first reported to induce *TNF* expression in monocyte‐derived macrophages and to promote tumour formation in *in vitro* and *in vivo* models (Yoshida et al. 1999; Suganuma et al. 2001). *H. pylori* secretes Tipα into culture supernatants during growth, with bacterial isolates from cases of gastric cancer found to secrete significantly greater amounts of this protein than those from gastritis cases (Suganuma et al. 2008).

Recombinant Tipα (rTipα) was shown to enter gastric epithelial cells, localise to the nuclear compartment and promote gastric carcinogenesis via the induction of epithelial‐mesenchymal transition (Watanabe et al. 2014). It was proposed that dimeric Tipα binds to cell‐surface nucleolin, which is typically expressed in the nucleolus, and is then internalised (Watanabe et al. 2010b). Upon localisation at the nucleus, Tipα binds to conserved regions of the *TNF* promoter upregulating gene transcription via NF‐κB (Suganuma et al. 2005; Kuzuhara et al. 2007). It is noteworthy, however, that the majority of these studies were performed using recombinant protein from the reference *H. pylori* 26695 strain. Furthermore, although Tipα possesses a signal sequence (Tosi et al. 2009), the mechanism by which the native protein may be secreted and delivered to host cells is not known.

Interestingly, Tipα was detected in the proteomes of *H. pylori* outer membrane vesicles (OMVs), referred to here as extracellular vesicles (EVs) (Turner et al. 2015). These spherical nanostructures (20–250 nm) are constitutively secreted by their parent bacteria (Kaparakis‐Liaskos and Ferrero 2015). Our group showed that bacterial EVs traffic intracellularly and localise to the nuclear compartment of epithelial cells (Bitto et al. 2017). Thus, we hypothesised that *H. pylori* EVs, which are potent regulators of intracellular innate immune signalling (Kaparakis et al. 2010; Irving et al. 2014), may be a novel mechanism by which the bacterium delivers Tipα to the host nucleus. Firstly, we confirmed that Tipα is secreted into culture supernatants during growth and showed that the amount secreted is strain‐dependent. Significantly, we demonstrated that Tipα is encapsulated within *H. pylori* EVs and, moreover, that the majority of secreted Tipα is associated with EVs and not present as free protein. Cell fractionation studies and confocal microscopy detected EV‐associated Tipα in the nuclear fraction of epithelial cells as early as 4 h after incubation with cells. Both EVs and their Tipα cargo were shown to bind genomic DNA. EVs from *H. pylori tipA* mutant bacteria induced greater pro‐inflammatory cytokine responses in host cells than wild‐type (WT) or complemented *tipA* (*tipA+*) bacteria, however, this finding was dependent on the EV isolation method. In conclusion, we have for the first time identified EVs as a novel mechanism for the secretion and delivery of a nucleomodulin to host cells.

## 2 Materials and Methods

### 2.1 Cell Lines and Culture Conditions

Gastric epithelial (AGS, MKN‐1, MKN‐74, TMK‐1, NUGC4 and N‐87) and THP‐1 cell lines were maintained in Roswell Park Memorial Institute (RPMI) medium (Gibco) supplemented with 10% (v/v) foetal bovine serum (Bovogen), 1% (v/v) L‐Glutamine (Gibco), 1% (v/v) HEPES (Gibco) with or without 1% (v/v) Penicillin/Streptomycin (Gibco). WT and *Tlr2^−/−^Tlr4^−/−^* immortalised C57/BL6 mouse macrophages (iMacs) (Bitto et al. 2017) were maintained in Dulbecco's Modified Eagle Medium (DMEM) (Gibco) supplemented as above. Cells were cultured in 5% CO_2_ at 37°C.

### 2.2 Bacterial Strains and Growth Conditions

The following strains were used: reference strain, 26695 (Akopyants et al. 1995); mouse‐colonising SS1 (Lee et al. 1997) and its clinical progenitor, 10700 (Lee et al. 1997) (more commonly known as PMSS1 (Arnold et al. 2011; Draper et al. 2017)); and mouse‐colonising B128 7.13 (Israel et al. 2001). These *H. pylori* strains and clinical isolates from cases of gastric cancer or functional dyspepsia were routinely cultured on Blood Agar no. 2 (HBA) base (Oxoid) or Brain Heart Infusion broth (Oxoid), supplemented with 7.5% (v/v) horse blood and Skirrow's selective supplement, as described previously (Ferrero et al. 1994). Media were supplemented with 20 µg/ml (w/v) kanamycin (Sigma‐Aldrich) and/or 10 µg/ml (w/v) chloramphenicol (Sigma‐Aldrich), as required. Bacteria were incubated at 37°C under microaerobic conditions (Campygen, Oxoid and Thermo Fisher Scientific). For plasmid construction and recombinant protein production, chemically competent *Escherichia coli* XL‐1 Blue or BL21 DE3 cells were cultured on Luria‐Bertani (LB) agar or broth supplemented with 100 µg/ml (w/v) ampicillin (Sigma‐Aldrich), 20 µg (w/v) kanamycin or 10 µg/ml (w/v) chloramphenicol, as appropriate.

### 2.3 Construction of *H. pylori tipA* and Complemented *tipA* (*tipA*+) Mutants

In brief, isogenic *tipA* mutants were constructed by insertional mutagenesis using a promoter‐less kanamycin cassette (*aphA3)* (Grubman et al. 2010). To construct complemented *tipA* (*tipA+*) mutants, a protocol was adapted from Langford et al. (2006). Briefly, the *tipA* gene including promoter region was PCR amplified from *H. pylori* 26695 with primers containing BamHI and SmaI restriction sites (Table S1). The amplified PCR product was inserted into the pIR203C04 plasmid to produce the pIR203tipAC04 complementation plasmid. Complemented *tipA* (*tipA+*) mutants were derived by natural transformation with either pIR203tipAC04 plasmid or genomic DNA from complemented *H. pylori tipA* (*tipA+*), as described previously (Langford et al. 2006). *H. pylori tipA* mutants and complemented *tipA* (*tipA+*) strains were confirmed by PCR, Sanger sequencing and Western blotting.

### 2.4 Mouse Colonisation Studies

Specific pathogen‐free mice (C57BL/6J, female) were purchased from the Walter and Eliza Hall Institute of Medical Research (WEHI; Melbourne, Australia). Animals were maintained at the Monash Health Translation Precinct Animal Facility (MHTPAF) under specific pathogen‐free conditions, in micro‐isolator cages with access to food and water ad libitum, under controlled temperature (18°C–22°C) and humidity (50%–60%) and a 12‐h dark/12‐h light cycle. Experiments were performed using female mice (4–6 weeks of age). Animal experimentation approval was obtained from by Animal Ethics Committee B(MMCB/2020/42, MMCB/2025/07). Mice were euthanised by CO_2_ inhalation.

Mice were inoculated once each via oral gavage with equivalent amounts of *H. pylori* strain SS1 WT, *tipA* and *tipA* (*tipA*+) bacteria (approximately 10^8^ colony‐forming units (CFUs)/mouse confirmed by agar plate dilution). Animals were euthanised at 1‐month post‐infection. Stomachs were homogenised in GentleMACS M tubes (Miltenyi Biotec), using a GentleMACS Disociator (Miltenyi Biotec) set to program ‘m_heart_0.2_01_C’. Stomachs were analysed for CFUs/g tissue by agar plate dilution, as described previously (Grubman et al. 2010). To detect gastric TNF, stomach homogenates were diluted 1:2 in DPBS and analysed by ELISA.

### 2.5 Production of rTipα Proteins

rTipα from *H. pylori* 26695 was produced using the pET‐Tipα plasmid, as described previously (Tosi et al. 2009). Protein was purified with Ni Sepharose 6 Fast Flow resin (GE Healthcare) as described by Tosi et al. (2009). To produce rTipα derived from *H. pylori* SS1, a DNA fragment corresponding to secreted Tipα starting at methionine 41 was amplified by PCR using primers containing BamHI and SacI restriction sites (Table S1). The amplified PCR product was inserted into the pET‐28a‐(c)+ vector to produce the pET‐SS1‐*tipA* plasmid. The resulting pET‐SS1‐*tipA* plasmid was confirmed by PCR and Sanger sequencing and SS1 rTipα produced and purified as above. For some experiments, the His‐tag was removed from rTipα preparations using a TEV‐protease (Sigma‐Aldrich) to generate a cleaved form of Tipα (cTipα).

### 2.6 Anti‐Tipα Serum Production

Rabbit polyclonal antiserum to rTipα derived from *H. pylori* 26695 was generated by the antibody facility at the WEHI. Rabbits were administered rTipα (200 µg) with complete Freund's adjuvant at day 1, then with rTipα and incomplete Freund's adjuvant at days 28 and 56.

### 2.7 EV Isolation and Purification

*H. pylori* EVs were isolated from bacterial culture supernatants by either ultracentrifugation or size exclusion chromatography (SEC). Ultracentrifuged (UC) EVs were isolated from *H. pylori* bacteria that had been inoculated in BHI broth (optical density_600_ = 0.05), supplemented with 6 mg/mL (w/v) β‐cyclodextrin (Sigma‐Aldrich) and Skirrow's selective supplement, and cultured for 16 h to mid‐exponential phase (Kaparakis et al. 2010) (Figure S1a). Culture supernatants were harvested by centrifugation at 3220 × *g* for 20 min at 4°C, then filter‐sterilised through 0.22 µm pore size filters (Corning). EVs were isolated from culture supernatants by ultracentrifugation at 136,400 × *g* for 2 h at 4°C. The concentrated EVs were then washed 3 times, each with 4 mL of Dulbecco's phosphate buffered saline (DPBS) (Gibco) and further concentrated using 10 kDa molecular weight cut‐off (MWCO) Amicon filters (Merck) and stored at −20°C.

For SEC, bacteria were grown in BHI broth depleted of non‐vesicular extracellular particles by tangential flow filtration (TFF) using a 50,000 kDa MWCO Viva flow (Sartorius), as described previously (Le et al. 2023). EVs were recovered from culture supernatants by ultracentrifugation at 136,000 × *g* for 2 h at 4°C, washed three times in 4 mL of DPBS, and concentrated using 100 kDa MWCO Amicon filters (Merck). EVs were subjected to SEC by loading onto a qEVoriginal 35 nm column (Izon), as per the manufacturer's instructions. The protein content of eluted fractions was determined using the Qubit Protein Assay with a Qubit 1.7 Fluorometer (Thermo Fisher Scientific), according to the manufacturer's guidelines. The EV containing fractions were pooled and concentrated using 100 kDa MWCO Amicon filters and stored at −20°C. As a control, BHI broth alone was treated as above.

### 2.8 EV Characterisation

Nanoparticle Tracking Analysis (NTA) was used to assess particle size distribution, median particle size and particle concentrations of EV preparations, using a PMX‐130 Zetaview instrument (Particle Metrix) (Table S2).

Transmission electron microscopy (TEM) was performed on EV preparations to analyse EV morphology and the presence of co‐isolating contaminants, using an FEI Tecnai Spirit or JEOL 1400Plus TEM (Le et al. 2023). Copper 300 mesh grids were first glow‐discharged at 30 mA for 30 s. EV samples (not fixed) were adhered to grids for 5 min at room temperature. The grids were washed three times in DPBS and then negatively stained with 2% (w/v) aqueous uranyl acetate for 10 s at room temperature. Grids were completely dried prior to imaging at a high‐tension voltage of 80 kV, with a beam current of 40 µA. Images were taken at ×15,000 and ×50,000 magnification. Three to five fields were imaged for each biological replicate of EVs.

The protein Qubit Assay (Thermo Fisher Scientific) was used to quantify the protein content of EV preparations. Each biological replicate of EVs was measured in duplicate.

EV surface proteins were removed by Proteinase K digestion, as described previously (Cvjetkovic et al. 2016; Le et al. 2024), with modifications. UC or SEC EVs (5 × 10^10^ particles/ml) were incubated with 20 µg/ml (w/v) Proteinase K (Thermo Fisher Scientific) for 1 h at 37°C. Proteinase activity was stopped by treatment with 5 mM phenylmethylsulphonyl fluoride (Thermo Fisher Scientific) for 10 min at room temperature.

### 2.9 Detection of Tipα in Cell Culture Supernatants

Free soluble and EV‐associated Tipα were obtained using a method adapted from Ricci et al. (2005). Briefly, culture supernatant was collected after sterile filtration and ultracentrifugation performed to generate samples of EV‐depleted supernatant (Figure S1b). EV pellets were recovered, washed with DPBS and ultracentrifuged. EV pellets were then resuspended to the original culture volume and sampled. Equivalent volumes of pre‐ultracentrifugation supernatant, EV‐depleted supernatant and resuspended EVs were collected for downstream analyses. Samples were concentrated by adding StrataClean resin beads (Agilent Technologies) and incubation overnight at 4°C. Concentrated samples were centrifuged for 10 min at 10,000 × *g* (4°C) and washed twice before resuspension in 50 mM Tris‐HCl (pH 8).

### 2.10 DiO Labelling of *H. pylori* EVs

Vybrant DiO Cell‐Labelling stain (Invitrogen) was added to *H. pylori* EVs or DPBS alone (1:100 dilution) and incubated in the dark for 30 min at 37°C. The EVs were then washed three times in PBS using 10 kDa molecular weight cut‐off Amicon filters (Merck) at 3220 × *g* for 30 min at 37°C each time, and stored at 4°C.

### 2.11 Electrophoretic Mobility Shift Assay (EMSA)

Total genomic DNA (gDNA) was extracted from AGS cells using the PureLink Genomic DNA Mini Kit (Invitrogen), as per the manufacturer's instructions. DNA was sonicated for two rounds of 30 s at 25% amplitude, alternating with a rest on ice for 30 s.

Sonicated DNA (250 ng) was mixed with rTipα protein (5 or 10 µg) and UC EVs from WT and *tipA* strains (1, 5 and 10 µg) and incubated at room temperature for 20 min prior to the addition of 6X gel loading dye with or without 0.01% (w/v) SDS (B7024S and B7025S, New England Biolabs). Prepared DNA‐protein complexes were separated on 6% polyacrylamide gels cast and run in 0.5 × TBE (65 mM tris [pH 7.6], 22.5 mM boric acid, 1.25 mM EDTA) at 200 V, for 45 min. DNA staining was performed using the Electrophoretic Mobility Shift Assay (EMSA) Kit (E33075, Invitrogen). Briefly, gels were submerged in 1X SYBR Green EMSA nucleic acid gel stain prepared in TBE, and incubated in the dark for 20 min with gentle agitation. The gels were washed three times in 150 ml distilled H_2_O to remove excess stain before imaging (Amersham Imager 680).

### 2.12 Cell Co‐Culture Assays

THP‐1 cells were seeded into 48‐well plates (2.5 × 10^5^ cells/ml, 0.4 ml/well) and differentiated for 48 h by adding 20 ng/ml (w/v) Phorbol 12‐myristate 13‐acetate (PMA, Sigma‐Aldrich) (Chonwerawong and Ferrero 2021). After 2 days, media were replaced and cells incubated for a further 2 days. AGS cells were seeded into 24‐well plates (1 × 10^5^ cells/ml, 1.0 ml/well) and incubated for 24 h. AGS cells were then serum‐starved for 24 h. WT and *Tlr2^−/−^Tlr4^−/−^* iMacs were seeded into 96‐well plates (1 × 10^5^ cells/ml, 0.1 ml/well) and incubated overnight.

Cells were co‐cultured with UC or SEC EVs (1 × 10^10^ particles/ml) or rTipα (unless stated otherwise, 50 µg/ml) for the indicated times. As controls, cells we co‐cultured with either BHI broth alone (negative) or Pam3CSK4 (1 µg/ml, w/v; InvivoGen; positive). For bacterial co‐culture assays, liquid cultures of *H. pylori* were added to cells at a multiplicity of infection (MOI) of 10:1, as described previously (Chonwerawong and Ferrero 2021). After 1 h, bacteria were removed, and cells were incubated in fresh media for the indicated times. Supernatants were harvested and stored at −20°C until analysed.

### 2.13 Cell Fractionation Assays

AGS cells were seeded in 6‐well plates (1.5 × 10^5^ cells/ml, 2.0 ml/well) and incubated for 24 h. The following day, *H. pylori* EVs (150 µg/ml) or rTipα (50 µg/ml) were added to cells and incubated for 4, 8 or 18 h. Cytoplasmic and nuclear fractions were isolated using a Nuclear Extraction Kit (Abcam), as per the manufacturer's instructions. The protein concentrations of the resulting fractions were determined by the Qubit Protein Assay (Thermo Fisher Scientific).

### 2.14 Western Blotting

To equalise protein loading, samples were quantified by the Qubit Protein Assay (Thermo Fisher Scientific). Samples were then resuspended in 4× Laemmli sample buffer (Bio‐Rad). For reducing and non‐reducing conditions, 355 mM 2‐mercaptoethanol (Merck) was either added or not, respectively. Samples were heated for 10 min at 100°C before loading on NuPage 4%–12% Bis‐Tris 1.5 mm gels (Invitrogen). Samples were electrophoresed for 10 min at 70 V, followed by 90 min at 120 V. Proteins were transferred onto nitrocellulose Amersham Protran Supported Western blotting membranes (Cytiva) for 70 min at 100 V, then stained with Ponceau S (Sigma‐Aldrich) to examine loading efficiency. Membrane blocking was performed in 5% (w/v) Bovine Serum Albumin (BSA) (Bovogen) in TBS‐Tween 20 (Sigma‐Aldrich) (0.05% v/v) for 1 h at room temperature. Membranes were incubated overnight at 4°C with either rabbit anti‐Tipα serum (1:5000), rabbit anti‐*H. pylori* EV serum (1:2000) (Kaparakis et al. 2010; Irving et al. 2014), rabbit anti‐*H. pylori* whole cell lysate serum (1:5000, (Ferrero et al. 1994)), mouse anti‐lamin A/C (1:1000, Cell Signalling Technologies), rat anti‐tubulin (1:2000, Abcam), rabbit anti‐Flotillin/HP0248 serum (1:1000, (Hutton et al. 2017)), rabbit anti‐HspA(GroES) (1:5000, (Suerbaum et al. 1994)), rabbit anti‐HspB(GroEL) (1:5000, (Suerbaum et al. 1994)), rabbit anti‐KatA (1:1000, Prof. Dianna Hocking, CSL Limited, unpublished), or rabbit anti‐UreB (1:2500, (Ferrero et al. 1994)) diluted in 3% (w/v) BSA in TBS‐Tween (0.05% v/v). Membranes were washed 3 times in TBS‐Tween (0.05% v/v) and incubated with either rabbit anti‐mouse IgG (H+L) horseradish peroxidase (HRP, 1:2000, Invitrogen), goat anti‐rabbit IgG (H+L) HRP (1:2000, Invitrogen), or goat anti‐rat IgG HRP (1:2000, Chemicon), diluted in 3% (w/v) BSA in TBS‐Tween (0.05% v/v) for 2 h at room temperature. Membranes were washed 3 times in TBS‐Tween and then developed with ECL Western Blotting Detection Reagents (Cytiva) and imaged using an Amersham Imager 680 (GE Healthcare).

### 2.15 Flow Cytometric Analysis of Surface‐Expressed Nucleolin

Gastric epithelial cell monolayers were washed in DPBS and incubated with 2 mM EDTA diluted in 1% (w/v) BSA, with gentle shaking to dissociate the monolayers. The Zombie UV Fixable Viability Kit (Biolegend) was used to stain for live and dead cells. Cells were blocked with TruStain FcX (1:250, anti‐mouse CD16/32) antibody (Biolegend) for 10 min on ice. Cells were washed with 2 mM EDTA diluted in 2% (w/v) BSA and then stained with mouse anti‐nucleolin antibody (1:250, Invitrogen) for 30 min at 4°C in the dark. Cells were washed as above and then incubated with anti‐mouse IgG Alexa Fluor 568 (1:1000 Invitrogen) secondary antibody. Cells were washed and then resuspended in 2 mM EDTA diluted in 2% (w/v) BSA, before analysis using a BD LSRFortessa X‐20 (BD Biosciences). A minimum of 10,000 events were collected for each cell line sample. The gating strategy involved sequential selection of single cell populations, containing viable cells that were nucleolin‐positive. The threshold for defining nucleolin‐positive cell populations was set using unstained cells specific to each cell line. Results were analysed using FlowJo software (Version 10; Treestar, Inc.).

### 2.16 Immunofluorescence and Confocal Microscopy

Gastric epithelial cells were seeded (2 × 10^5^ cells/ml, 0.2 ml/well) in 8‐well Ibidi chambers (Ibidi) and incubated for 24 h. Cells were incubated with either *H. pylori* EVs or 26695 rTipα (all 50 µg/ml) and incubated for the appropriate times. Cells were washed with DPBS. To inhibit endocytosis or macropinocytosis, cells were washed twice and then treated with Dynasore (10 µM; Sigma‐Aldrich) or Cytochalasin D (2 µM; Sigma‐Aldrich), respectively (Turner et al., 2018). As a control, cells were treated with the vehicle, dimethyl sulphoxide (DMSO). After 30 min incubation at 37°C, cells were washed twice with DPBS prior to EV addition. Background autofluorescence was quenched by incubating cells in 0.025% (w/v) trypan blue (Gibco) for 4 min, followed by washing. Cells were fixed in 4% (w/v) paraformaldehyde for 10 min at room temperature, followed by DPBS wash. For permeabilised conditions, cells were incubated with blocking buffer comprising 3% (w/v) BSA diluted in 0.1% (v/v) Triton X‐100 for 1 h at room temperature. For non‐permeabilised conditions, Triton X‐100 was omitted from the blocking buffer. After blocking, cells were incubated with either mouse anti‐nucleolin (1:500, Abcam), rabbit anti‐Tipα serum (1:200), mouse anti‐GM130 (1:200, BD Biosciences), mouse anti‐calnexin (1:100, Invitrogen), rabbit pre‐bleed serum (1:100), or mouse IgG isotype control (1:100, Dako) overnight at 4°C. Cells were washed in blocking buffer and then incubated with either anti‐mouse IgG Alexa Fluor 568 (1:500) or anti‐rabbit IgG Alexa Fluor 488 (1:500, Invitrogen) for 2 h in the dark at room temperature. Cells were then washed as above and incubated in Hoescht (1:2000, Invitrogen) for 5 min at room temperature in the dark. Cells were washed and then mounted using Dako mounting medium (Agilent) and imaged on either the EVOS M5000 (Thermo Fisher Scientific) or a Nikon C1 inverted confocal microscope.

### 2.17 Co‐Immunoprecipitation

Gastric epithelial cells were seeded (2 × 10^5^ cells/ml, 2.0 ml/well) in 6‐well plates and incubated overnight. Cells were then incubated with *H. pylori* 26695 rTipα in which the His‐tag had either been cleaved (cTipα) or not (both 50 µg/ml), for 1 h or 18 h, respectively. Cells were then washed twice with ice‐cold DPBS. Proteins were cross‐linked with 2.25 mM disuccinimidyl suberate (Thermo Fisher Scientific) for 30 min at room temperature. After incubation, cross‐linking was stopped by the addition of 20 mM Tris‐HCl (final concentration) and incubation for 15 min at room temperature. Cells were washed with ice‐cold DPBS and then lysed using NP‐40 lysis buffer (Thermo Fisher Scientific). Lysates were aspirated and then centrifuged at 15,700 × *g* for 15 min at 4°C. Lysates were left untreated, incubated with anti‐Tipα (1:2000) or pre‐bleed (1:4800) sera and incubated for 1 h at 4°C. Pierce Protein A/G Magnetic Beads (Thermo Fisher Scientific) were added to lysates and incubated with rolling for 3 h at 4°C. Samples were then washed twice with ice‐cold DPBS prior to Western blotting.

### 2.18 ELISA

Human TNF, IL‐8 and mouse TNF levels were quantified by ELISA, as per the manufacturer's instructions (BD Biosciences). Absorbance values were measured at 450 nm with a CLARIOstar microplate reader (BMG Labtech). Cytokine levels were determined by 4‐parameter fit analysis. Serum samples from animal infection studies were analysed as previously described (Ferrero et al. 1995). In brief, serum was diluted 1:100 (v/v) and added to Nunc Maxisorb ELISA plates coated with 25 µg *H. pylori* sonicate.

### 2.19 In Silico Multiple Sequence Alignments

The genomes of gastric and enterohepatic *Helicobacter* species were identified in NCBI's Genome resource (Table S3). Species which encoded *tipA* were identified by performing BLASTN analyses with *H. pylori* 26695 *tipA* (HP_RS02940) as the query sequence and the identified genomes as the subject sequences. Clustal Omega multiple sequence alignment was then performed to align the translated Tipα amino acid sequences (Madeira et al. 2024). Percentage protein identity to *H. pylori* 26695 Tipα (Table S3), amino acid sequence alignment (Figure S2a), and phylogenetic tree (Figure S2b) were derived from Clustal Omega outputs.

### 2.20 Statistical Analysis

GraphPad Prism version 10.4.0 (GraphPad Software) was used for graphical presentation and statistical analysis. Data was analysed by ordinary one‐way ANOVA with Tukey's multiple comparisons test or two‐way ANOVA with multiple comparisons, as indicated. *P* values <0.05 were considered statistically significant.

## 3 Results

### 3.1 Tipα is Highly Conserved Amongst *H. pylori* Isolates and Gastric Helicobacter spp

Current knowledge regarding *H. pylori* Tipα is based on structure‐function studies using recombinant forms of the protein derived from the reference 26695 strain. To gain a broader understanding of *H. pylori* Tipα, we performed *in silico* analyses on the homologues from several common laboratory strains, as well as a selection of closely related gastric *Helicobacter* spp. We found that Tipα is ubiquitously found in the species and highly conserved, sharing 94.27%–98.96% identity at the protein level with Tipα from the 26695 strain (Table S3). A notable exception, however, was the presence of a 20‐amino acid extension to the signal peptide sequence, which was reported in a subset of strains, including the mouse‐adapted *H. pylori* SS1 strain, its parental clinical isolate, PMSS1, and two other common laboratory strains (G27, X47‐2AL) (Figure S2a). As reported previously (Godlewska et al. 2008; Morningstar‐Wright et al. 2022), we found that *H. pylori* SS1 *tipA* bacteria were unable to colonise mice (Figure S3a). Consistent with the role of Tipα as an inducer of TNF responses (Yoshida et al. 1999; Suganuma et al. 2001), we also showed that gastric mucosal levels of TNF in the Tipα‐challenged mice were lower, albeit not significantly different, to those in animals infected with WT bacteria (Figure S3b). Serum IgG levels were also reduced in mice that received *tipA* bacteria, when compared with WT‐infected animals, while no changes in serum IgA levels were observed between the groups (Figure 3c,d).

Tipα homologues are present in all closely related gastric *Helicobacter* species (36.31%–89.58% protein identity vs. *H. pylori* 26695 Tipα) but absent from all other bacterial genera (data not shown). Tipα homologues were also absent in the ferret gastric *Helicobacter* spp., *Helicobacter mustelae*, and *Helicobacter* spp. that infect the colon or hepatobiliary system (Table S3). Interestingly, it was previously observed that *H. mustelae* also differed from other gastric *Helicobacter* spp. in lacking amidase and deamidase‐encoding genes (Bury‐Moné et al. 2003; Leduc et al. 2010). This finding was attributed to *H. mustelae* being phylogenetically closer to enterohepatic *Helicobacter* spp. by 16srRNA sequencing (Solnick and Schauer 2001). Taken together, we propose that Tipα is an evolutionarily conserved trait amongst *Helicobacter* spp. that colonise the stomach.

### 3.2 The Amount of Tipα Secretion Is Strain‐Specific

**FIGURE 1:**
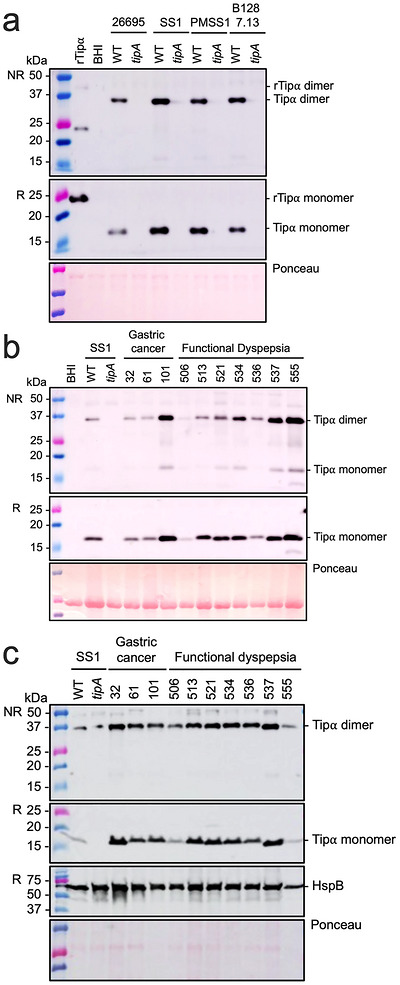
**The amount of Tipα secreted is strain‐specific but not associated with disease outcome**. (a, b) Bacterial culture supernatants and (c) whole cell lysates were analysed by Western blotting using anti‐Tipα serum. (a) Culture supernatants from *H. pylori* WT and *tipA* mutant bacteria generated in strains: 26695, SS1, PMSS1 and B128 7.13. (b) Culture supernatants and (c) whole cell lysates from *H. pylori* isolates associated with either gastric cancer or functional dyspepsia. The housekeeping protein HspB (GroEL) was detected using antiserum to confirm the equivalent loading of samples. Ponceau red stain was used to detect total proteins. NR = non‐reducing conditions, R = reducing conditions. Molecular weight markers are indicated on the left of each figure panel. Images are representative of independent experiments: (a) *n* = 2, (b) *n* = 3 or (c) *n* = 1.

We next studied Tipα secretion in culture supernatants from a range of *H. pylori* laboratory and clinical isolates. Consistent with previous reports (Suganuma et al. 2005; Suganuma et al. 2008), we detected both dimeric and monomeric forms of Tipα in culture supernatants of these bacteria (Figure 1a,b). To confirm the findings, we generated *tipA* mutants on multiple strain backgrounds (Figure 1a,b). Furthermore, we restored Tipα production in *tipA* mutants by genetic complementation, with *tipA* (*tipA+*) strains producing comparable levels of the protein to the parental wild‐type (WT) bacteria (Figure S4). Significantly, we observed strain‐specific differences in the amounts of Tipα secretion amongst *H. pylori* strains. Contrary to previous findings (Suganuma et al. 2005; Suganuma et al. 2008), however, it did not appear that isolates from gastric cancer cases secreted greater amounts of the protein when compared with those associated with functional dyspepsia (Figure 1b). SDS‐PAGE analysis of whole cell preparations of these isolates indicated that similar amounts of Tipα are produced by the bacteria (Figure 1c), implying that the varying amounts of protein in supernatants may be attributed to differences in secretion. *H. pylori* SS1 and PMSS1 strains both have *tipA* sequences that include the extended signal peptide sequence, yet the bacteria secreted different amounts of dimeric Tipα (Figure 1a). Also, the amount of Tipα secreted by *H. pylori* PMSS1 was more comparable to that released by *H. pylori* B128 7.13, in which *tipA* has the shorter signal peptide sequence, suggesting that it is unlikely that the levels of Tipα secretion correlate with the differences in sequence. Taken together, *H. pylori* bacteria secrete Tipα in a strain‐specific manner. Due to the limited number of clinical isolates analysed, however, it is not possible to reach a conclusion regarding correlations between Tipα secretion by *H. pylori* isolates and disease outcome. Thus, further investigation with a greater number of clinical isolates is warranted.

### 3.3 *H. pylori* EVs Contain Tipα

**FIGURE 2:**
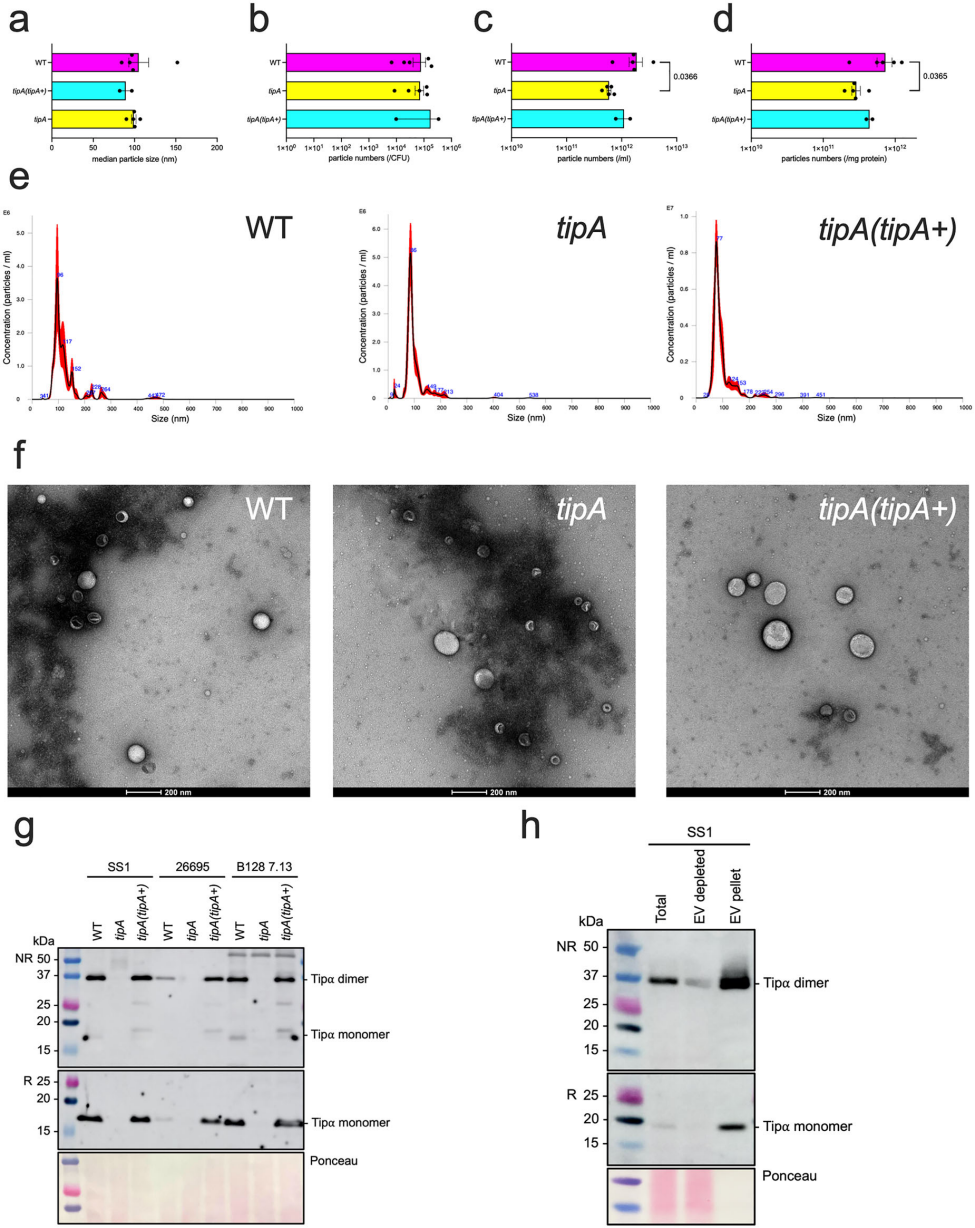
**Characterisation of Tipα in *H. pylori* UC EVs and supernatants**. (a–e) NTA on EVs isolated by ultracentrifugation from *H. pylori* SS1 WT, *tipA* mutant and complemented *tipA* (*tipA+*) bacteria. UC EV preparations were analysed for their (a) median particle sizes and numbers (b) per CFU, (c) per ml, (d) per mg of protein, as well as (e) the size distributions of these particles. (f) TEM images of the particles in each preparation (× 26,000 magnification, scale bar = 200 nm). (g) Western blotting of UC EVs isolated from *H. pylori* WT, *tipA* mutant, complemented *tipA* (*tipA+*) bacteria generated in strains: SS1, 26695 and B128 7.13. (h) Western blotting of supernatant prior to ultracentrifugation, EV‐depleted broth culture supernatant or resuspended EV pellet isolated from *H. pylori* SS1. Ponceau red stain was used to detect total proteins. NR = non‐reducing conditions, R = reducing conditions. Molecular weight markers are indicated on the left of each figure panel. NTA data for *H. pylori* SS1 WT and *tipA* mutant UC EVs (*n* = 5 independent preparations) and complemented *H. pylori* SS1 *tipA* (*tipA+*) (*n* = 2 independent preparations). Data correspond to the means ± standard error of the mean (SEM). Statistical analyses were determined by the unpaired t test. Images are representative of: (g) *n* = 1 and (h) *n* = 3 independent experiments.

Tipα contains a signal peptide sequence (Suganuma et al. 2005; Godlewska et al. 2008), suggesting that it may be released via the general secretion pathway. Nevertheless, as we detected Tipα in the proteome of *H. pylori* EVs (Turner et al. 2015), we hypothesised that the protein may also be secreted with EVs. To address this hypothesis, we first purified and characterised EVs by UC from three *H. pylori* WT strains, as well as their corresponding isogenic *tipA* and complemented *tipA* (*tipA+*) mutant bacteria. EVs were isolated from *H. pylori* broth culture supernatants by ultracentrifugation and then dialysed and further concentrated with 10 kDa Amicon MWCO filters. NTA was performed to characterise the particle size, particle numbers, particles produced per CFU and particles per mg of protein content. The median sizes and numbers of *H. pylori* SS1 EV particles produced per CFU were similar between preparations of WT and mutant strains (Figure 2a,b). Interestingly, EV preparations from *H. pylori tipA* mutant bacteria contained significantly fewer particles relative to volume and protein content, as compared with those of the corresponding WT bacteria (Figure 2c,d). EVs from *H. pylori* SS1 WT, *tipA* mutant and complemented *tipA* (*tipA+*) mutant bacteria did not, however, differ in their size distributions (Figure 2e). TEM analysis of SS1 UC EV particles showed the presence of typical spherical vesicle structures and did not contain co‐isolating bacterial products, such as flagella (Figure 2f, Figure S5). Importantly, EV preparations from *H. pylori* SS1, 26695 and B128 7.13 WT and complemented *tipA* (*tipA+*) bacteria, but not *tipA* mutant bacteria, contained Tipα protein (Figure 2g).

Next, we sought to determine the contribution of EVs to Tipα secretion. For this, we modified the EV isolation protocol (Figure S1a) by first depleting culture supernatants of EVs via ultracentrifugation, then comparing the amounts of Tipα present in supernatants with those in the pellets recovered after ultracentrifugation (Figure S1b). Interestingly, very little free soluble Tipα was secreted into culture supernatants, with a majority of the protein associated with EVs (Figure 2h). Thus, we propose that EVs represent a major mechanism by which *H. pylori* secretes Tipα.

### 3.4 Influence of the EV Isolation Method on *H. pylori* EV Characteristics

**FIGURE 3:**
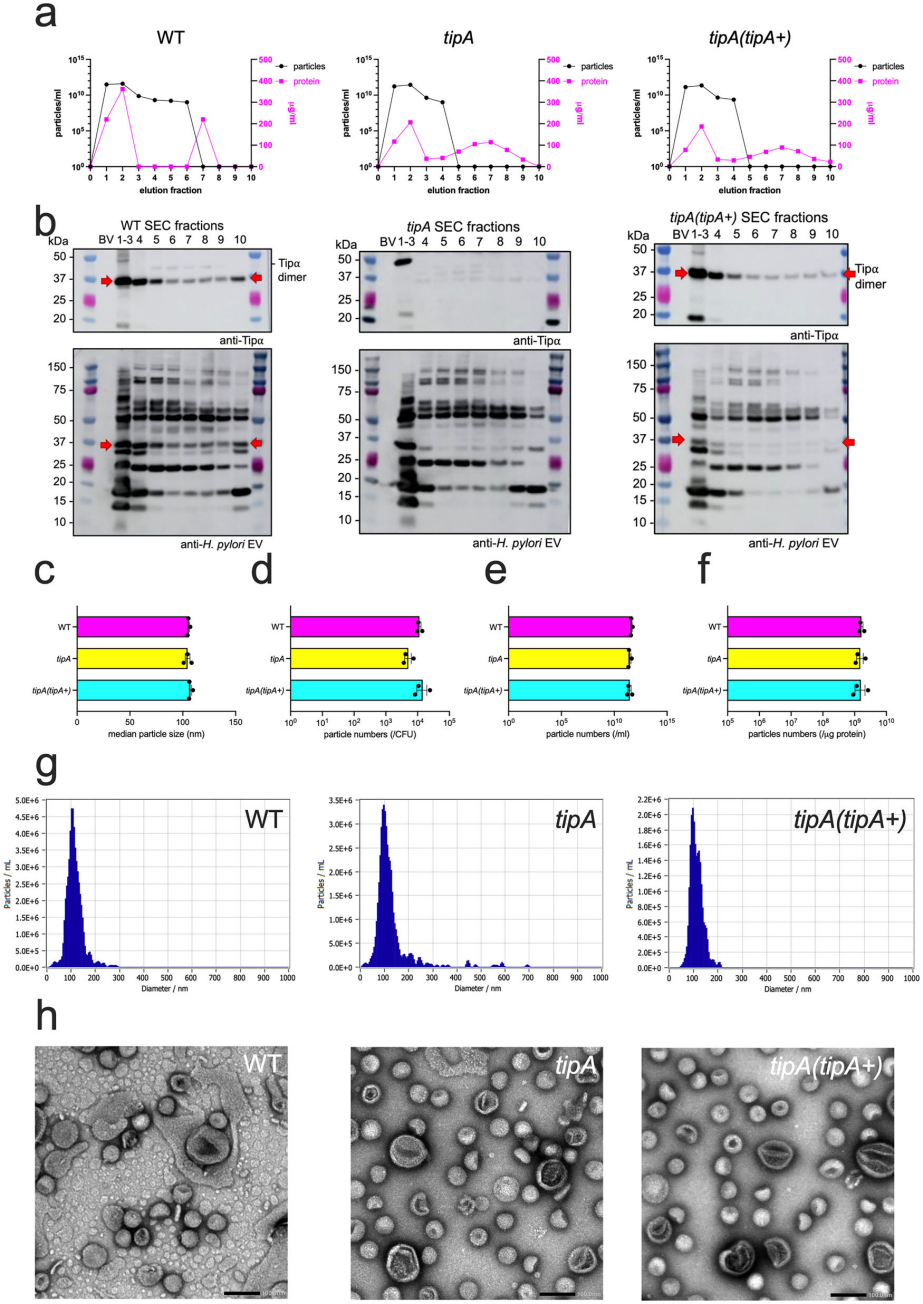
**Characterisation of *H. pylori* SS1 WT, mutant *tipA* and complemented *tipA (tipA+)* EVs isolated by SEC**. (a–c) NTA (particles/ml) and protein content (µg/ml) analysis of purified fractions after SEC. (b) Western blotting of purified fractions after SEC. Blots were reacted with either anti‐Tipα (top) or anti‐*H. pylori* EV (bottom) antisera. Samples were analysed under non‐reducing conditions. Molecular weight markers are indicated on the left of each figure panel. Red arrows indicate the location of dimeric Tipα. Yellow lines indicate a reference protein present in all strains. (c–f) NTA on SEC EVs from *H. pylori* SS1 WT, *tipA* mutant and complemented *tipA (tipA+)* bacteria. SEC EV preparations were analysed for their: (c) median particle sizes and numbers (d) per CFU, (e) per ml, (f) per mg of protein, as well as (g) the size distributions of these particles. (h) TEM images of the particles in each SEC EV preparation (× 50,000 magnification, scale bar = 100 nm). Data correspond to technical triplicates presented with the mean ± standard error of the mean (SEM).

The method used to isolate EVs has been shown to have an impact on EV characteristics (Takov et al. 2019). To address this potential issue, we isolated EVs using SEC from *H. pylori* SS1 WT, *tipA* mutant and *tipA (tipA+)* bacteria. SEC fractions were examined for particle number by NTA and by protein assay (Figure 3a), as well as by Western blotting using anti‐Tipα and ‐*H. pylori* EV antisera (Figure 3b). Like UC EV preparations, WT and complemented *tipA (tipA+)* SEC EV preparations contained Tipα, but *tipA* mutant SEC EVs did not (Figure 3b; Figure S6). Tipα was also detected in all other SEC fractions, indicating that a substantial amount of Tipα was dissociated from the EVs during SEC. In addition, several bands that were detected in the pooled SEC EV samples with *H. pylori* EV antiserum were also detected in other SEC fractions, suggesting the separation of other EV‐associated components (Figure 3b).

Most EV particles were detected in SEC fractions 1–3 (Figure 3a), which were pooled and analysed further. EVs isolated by SEC shared similar particle sizes and numbers isolated per CFU to those isolated by UC (Figure 3c–d). In contrast, EVs isolated by SEC were more consistent in terms of the number of particles relative to volume and protein content than were those isolated by UC (Figure 3e, f; Figure S6a–e). SEC EVs also had a uniform size distribution (Figure 3g), lacked the presence of co‐isolating bacterial products, like flagellar filaments (Figure 3h), and had slightly greater amounts of Tipα per particle than UC EVs although this did not reach statistical significance (Figure S6d,e).

**FIGURE 4:**
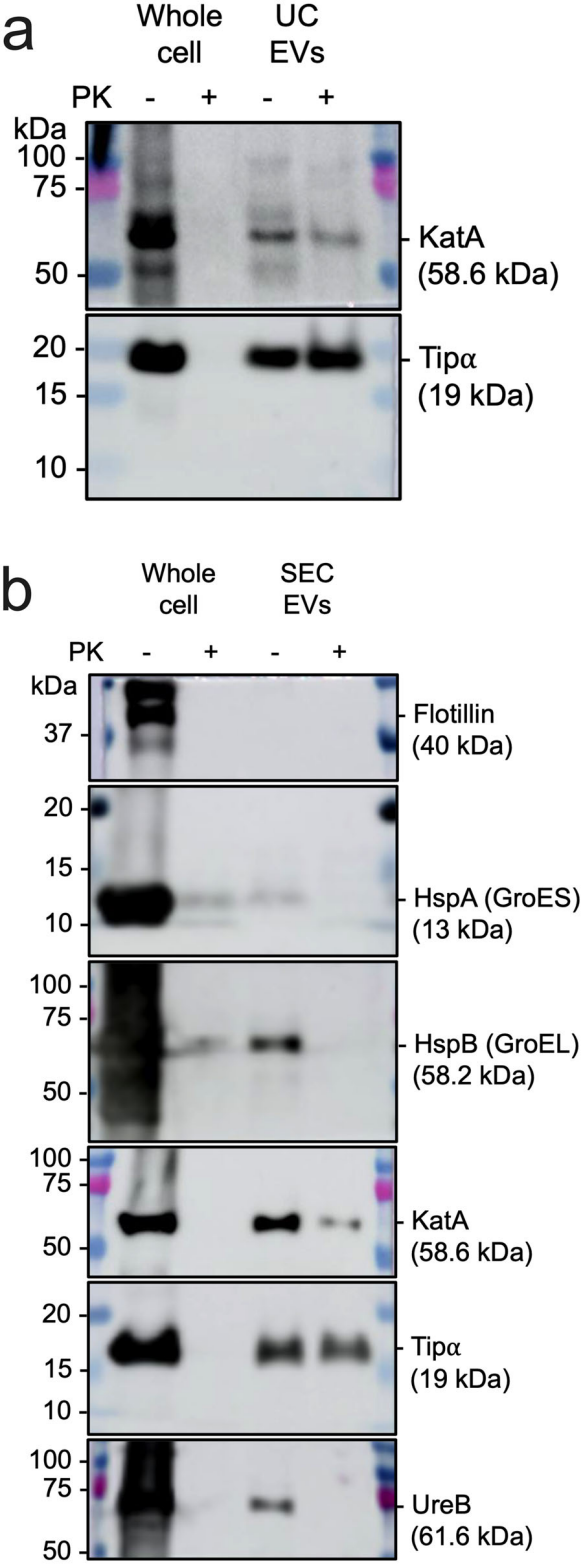
***H. pylori* Tipα is mainly located within EVs**. Surface‐associated proteins on EVs were digested using proteinase‐K (PK), or not, for 1 h at 37°C. Western blotting was performed on (a) UC and SEC EVs with antibodies directed against Flotillin (HP0248), HspA (GroES), HspB (GroEL), KatA, Tipα or UreB. As a digestion control, bacterial whole cells were also treated with proteinase‐K. Western blots were performed under reducing conditions. Images for UC and SEC EVs are representative of *n* = 2 and *n* = 3 independent preparations, respectively.

To assess the localisation of EV‐associated Tipα, we performed Western blotting on UC‐ and SEC‐purified EVs that had been subjected or not to proteinase‐K digestion. As can be seen, Tipα could be detected in both proteinase‐K‐treated and untreated EVs from UC and SEC preparations, whereas it was digested in bacterial whole cell preparations (Figure 4, Figure S6f–h). In contrast, proteinase‐K treatment of EV preparations resulted in the digestion of several *H. pylori* proteins that were reported in proteomic studies to be EV‐associated that is, heat shock proteins HspA (GroES) and HspB (GroEL), catalase (KatA) and urease subunit B (UreB) (Mullaney et al. 2009; Olofsson et al. 2010; Turner et al. 2015; Turner et al. 2018; Wei et al. 2022; Zavan et al. 2019) (Figure 4). Interestingly, we were unable to detect *H. pylori* flotillin (HP0248), which belongs to a family of proteins typically associated with the cytoplasmic membrane (Hutton et al. 2017). This suggests that *H. pylori* flotillin may be a non‐EV‐associated marker and, moreover, that there is specific packaging of the protein cargo in EVs, as reported by Haurat et al. (2011).

In conclusion, the purification of EVs by SEC improved the uniformity of the preparations across WT and mutant *H. pylori* strains but also likely resulted in the dissociation from EVs of some protein content, including Tipα. Taken together, the findings strongly indicate that Tipα is mainly encapsulated within *H. pylori* EVs.

### 3.5 EVs Carrying Tipα Localise to the Nuclear Compartment and Bind DNA

**FIGURE 5:**
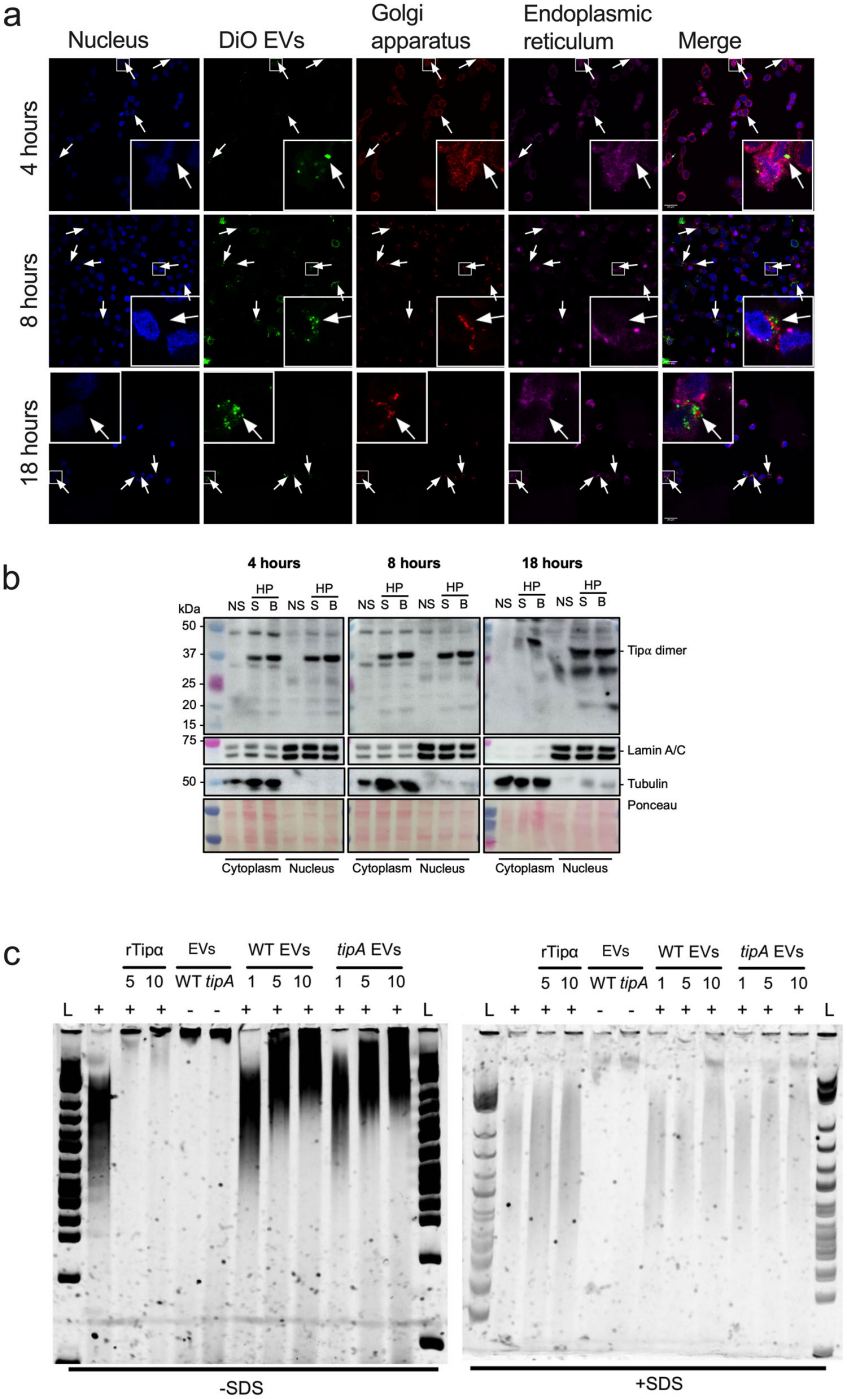
**EV‐associated Tipα localises to the nuclear compartment of gastric epithelial cells**. (a) AGS cells were incubated with DiO‐labelled *H. pylori* EVs for 4–18 h. Intracellular compartments are shown as follows: Golgi apparatus (red), endoplasmic reticulum (magenta) and nuclei (blue). Images: 60× magnification, scale bars = 20 µm. Arrows indicate *H. pylori* EVs localised in the perinuclear region. (b) AGS cells were cultured with EVs from either *H. pylori* SS1 or B128 7.13 WT bacteria, or left unstimulated, for 4–18 h before the fractionation of cytoplasmic and nuclear contents. Fractioned samples were analysed by Western blotting against anti‐Tipα serum on non‐reducing gels. Anti‐Lamin A/C and anti‐tubulin antibodies were used as markers for the nuclear and cytoplasmic compartments, respectively. Ponceau red stain was used to detect total proteins. Molecular weight markers are indicated on the left of each figure panel. (c) EMSA was performed to determine the DNA‐binding activity of rTipα (5, 10 µg) and EVs from WT and *tipA* strains (1, 5, 10 µg). Samples were pre‐incubated with 250 ng of sonicated gDNA from AGS cells (indicated by “+”), prior to electrophoresis on a 6% polyacrylamide gel in 0.5× TBE at 200 V for 45 min, with or without 0.01% (w/v) SDS added. DNA was visualised by staining with 1X SYBR Green for 20 min. L: 1 kb plus ladder (N3200, New England Biolabs). Images are representative of: (a) *n* = 1, (b) *n* = 3 and (c) *n* = 3 independent experiments.

Given that EVs are highly efficient at entering epithelial cells and trafficking to the perinuclear regions of host cells (Bitto et al. 2017), we reasoned whether this may be a mechanism by which Tipα traffics to the nucleus. To address this, time‐course experiments were performed in which the trafficking of DiO‐labelled *H. pylori* EVs in AGS gastric epithelial cells was assessed by confocal microscopy. DiO‐labelled EVs could already be observed close to the nuclei after 4 h of incubation with AGS cells and not associated with the Golgi apparatus or endoplasmic reticulum, thus confirming *H. pylori* EVs can reach the nucleus (Figure 5a). These findings were supported by cell fractionation studies (Figure 5b) and by experiments in the MKN‐1 gastric epithelial cell line (Figure S7). Further evidence for EV internalisation and localisation to the perinuclear region in host cells was provided by the pre‐treatment of cells with Dynasore and Cytochalasin D, pharmacological inhibitors of dynamin‐dependent endocytosis and micropinocytosis, respectively (Figure S8, Supplementary Videos S1, 2). These are two of the major pathways required for bacterial EV entry into host cells (Turner et al. 2018). Consistent with a previous report (Turner et al. 2018), the inhibitors reduced EV cell entry by approximately 50% and, consequently, also perinuclear localisation (Figure S8).

Next, we sought to determine whether *H. pylori* EVs might bind DNA by EMSA. We were able to show a distinct shift in gel migration when increasing amounts of gDNA were mixed with EVs (Figure 5c). DNA was detected in the EV preparations, which is consistent with our previous findings (Bitto et al. 2017). As reported by other workers (Suganuma et al. 2005; Kuzuhara et al. 2007), we showed that rTipα can directly bind DNA, however, the absence of Tipα from EVs had no effect on DNA binding (Figure 5c). Importantly, DNA binding by EVs and their Tipα cargo could be abrogated by the addition of SDS, thereby showing that this binding was specific (Figure 5c).

**FIGURE 6:**
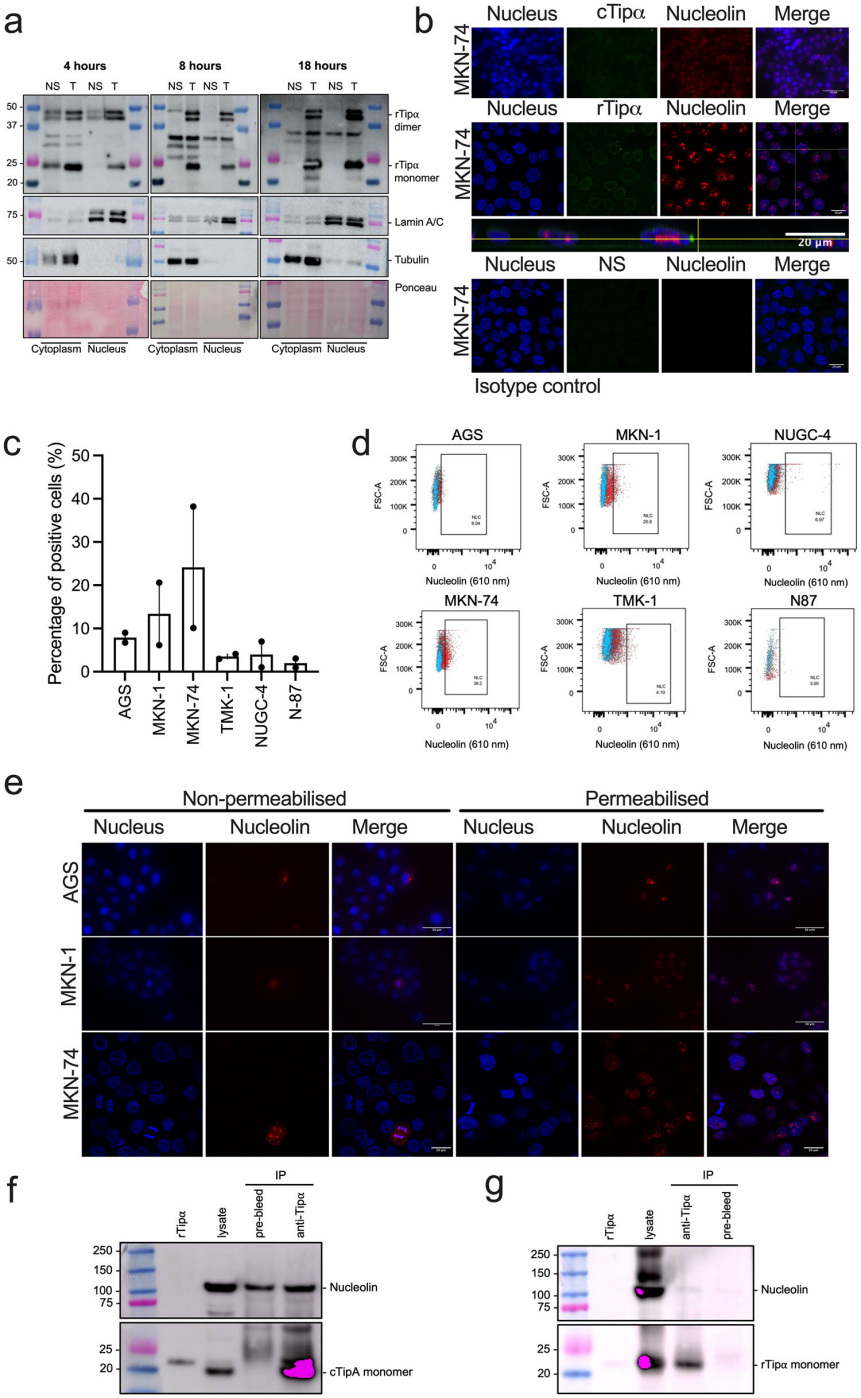
**rTipα binding to nucleolin binding is dispensable for its trafficking to the nucleus**. (a) AGS cells were incubated with rTipα (T; 50 µg/ml), or left untreated (NS), for 4–18 h before fractionation into cytoplasmic and nuclear compartments. Fractionated samples were analysed by Western blotting against anti‐Tipα serum. Anti‐lamin A/C and anti‐tubulin antibodies were used as markers for nuclear and cytoplasmic fractions, respectively. Ponceau red stain was used to detect total proteins. Molecular weight markers are shown on the left of each figure panel. (b) Flow cytometric analysis of surface nucleolin expression in gastric cancer cell lines: AGS, MKN‐1, MKN‐74, TMK‐1, NUGC‐4 and N‐87. Expression is reported as the percentages of positive cells. (c) Gating strategy for the detection of surface nucleolin. (d) Confocal microscopy on cell lines most highly expressing surface nucleolin (AGS, MKN‐1 and MKN‐74), showing nucleolin (red) and nuclei (blue), under either non‐permeabilised or permeabilised conditions, to detect surface and nucleolar nucleolin, respectively. Images: 20× or 60× magnification (scale bars = 50 or 20 µm). Confocal microscopy on (e) MKN‐74 cells that had been incubated with cTipα or rTipα (both 50 µg/ml) for 18 h, showing cTipα or rTipα (green), nucleolin (red) and nuclei (blue). Images: 20× or 60× magnification, scale bars = 50 or 20 µm. As a control, cells were reacted with a mouse IgG antibody isotype control. (f) MKN‐74 cells were incubated with rTipα (50 µg/ml) for 18 h before lysates were prepared for co‐immunoprecipitation. Lysates were co‐immunoprecipitated using anti‐Tipα serum or pre‐bleed serum as a control. Total lysate and immunoprecipitated lysates were probed using antisera to nucleolin or Tipα. (g) MKN‐74 cells were incubated wcTipα (50 µg/ml) for 1 h before lysates were prepared and co‐immunoprecipitation performed as per (f). Flow cytometry data are presented as the means ±SEM and representative of 2 independent experiments. Immunofluorescence, nuclear fractionation and co‐immunoprecipitation experiments were each performed once.

rTipα was reported to localise to the perinuclear region of gastric epithelial cells and that binding to surface nucleolin was required for its entry (Suganuma et al. 2008). Indeed, we detected rTipα in nuclear fractions (Figure 6a,b), however, contrary to previous work (Watanabe et al. 2010b), nucleolin was found to be poorly expressed across several gastric cancer cell lines (Figure 6c–e). Furthermore, co‐immunoprecipitation experiments with the anti‐rTipα antibody did not confirm rTipα binding to nucleolin (Figure 6f,g), thus we concluded that nucleolin binding was dispensable for Tipα localisation to the nucleus. Taken together, the data demonstrate that *H. pylori* EVs are able to deliver their Tipα cargo to the nuclear compartment of host cells and we suggest that this is likely to occur in a nucleolin‐independent manner.

### 3.6 Effects of EV‐Associated Tipα on Proinflammatory Cytokine Responses

**FIGURE 7:**
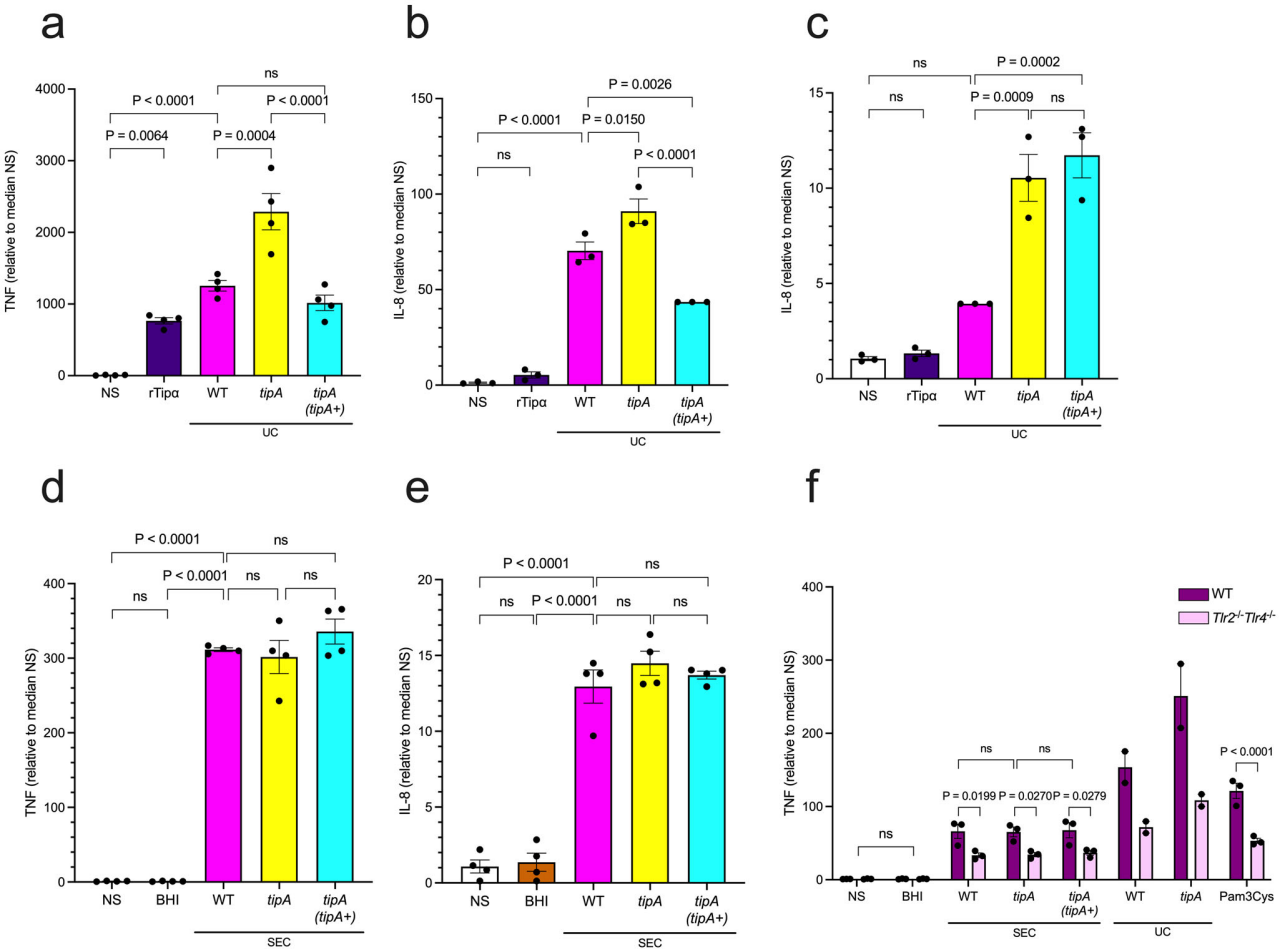
**Effects of EV‐associated Tipα on proinflammatory cytokine responses in host cells**. (a–c) AGS and THP‐1 cells were incubated with UC EVs from either *H. pylori* SS1 WT, *tipA* mutant, or complemented *tipA* (*tipA+)* bacteria, corrected to 10^10^ EV particles. As a positive control, rTipα (50 µg/ml) was added to cells. Detection of (a) TNF produced by THP‐1 cells (at 6 h post‐incubation) and (b) IL‐8 produced by THP‐1 cells and (c) AGS cells (at 24 h post‐incubation). (d, e) THP‐1 cells were incubated with 1 × 10^10^/ml SEC EVs from either *H. pylori* SS1 WT, *tipA* mutant, or complemented *tipA (tipA+)* bacteria. As a negative control, an equivalent volume BHI was added to cells. Detection of (d) TNF produced by THP‐1 cells (at 6 h post‐incubation) and (e) IL‐8 by THP‐1 cells (at 24 h post‐incubation). (f) WT and *Tlr2^−/−^Tlr4^−/−^* iMacs were incubated with 1 × 10^10^/ml SEC or UC EVs. As a negative control, an equivalent volume of BHI was added to cells. As a positive control, 1 µg/ml Pam3CSK4 was added to cells. TNF production was detected at 6 h post‐incubation. Each data point corresponds to the mean of 3 technical replicates from one independent experiment and normalised relative to the median value for NS cells. Data were combined from (a–e) 3–4 and (f) 2–3 independent experiments. Histograms correspond to the means ± SEM. Statistical analyses were determined by either (a–e) one‐way ANOVA with Tukey's multiple comparisons or (f) two‐way ANOVA with multiple comparisons.

Once localised to the nucleus, rTipα was reported to promote tumorigenesis by inducing *Tnf* expression in mouse gastric epithelial cell line, MGT‐40 (Suganuma et al. 2005; Suganuma et al. 2008), and TNF production in human peripheral blood monocytes (Yoshida et al. 1999). Consistent with previous work using human monocytes (Yoshida et al. 1999), we showed that rTipα from the *H. pylori* 26695 strain induces significantly higher levels of TNF production in THP‐1 cells, as compared with unstimulated cells (Figure 7a). Interestingly, rTipα generated from *H. pylori* SS1 also induced TNF (and IL‐8) production in THP‐1 cells, but these responses were significantly lower than those induced by rTipα from 26695 (Figure S9). It is unlikely that the differences in responses were due to endotoxin contamination, as the protein preparations had comparable levels (approximately 20–30 ng) of lipopolysaccharide (LPS). Instead, we propose that the varying levels of immunogenicity of the two recombinant proteins may be attributed to differences in their primary amino acid sequences (Figure S2) and deserves further investigation.

We next determined the effect of EV‐associated Tipα on inflammatory responses by culturing human THP‐1‐derived macrophages and AGS gastric epithelial cells with UC EVs from either *H. pylori* WT, *tipA* mutant or complemented *tipA* (*tipA+*) bacteria. rTipα from *H. pylori* 26695 was used as a positive control. Contrary to the findings for purified rTipα (Figure 7a), *H. pylori tipA* mutant UC EVs induced significantly greater TNF responses than EVs from either WT or complemented *tipA* (*tipA+*) bacteria (Figure 7a). This result suggests potential downregulation of TNF responses by Tipα when associated with UC EVs. As for TNF responses, rTipα also induced higher levels of the pro‐inflammatory cytokine, IL‐8, but the differences were not statistically significant (Figure 7b). Importantly, we observed increased IL‐8 responses to UC EVs from *tipA* mutant bacteria when compared with those from either WT or complemented *tipA* (*tipA+*) bacteria (Figure 7b). IL‐8 responses to both rTipα and UC EVs in AGS gastric epithelial cells followed similar trends as for those in THP‐1‐derived macrophages (Figure 7c). TNF responses were not measured in AGS cells as its production is undetectable in this cell line (unpublished data). Inexplicably, in AGS cells, UC EVs from complemented *H. pylori tipA* (*tipA+*) bacteria did not dampen cytokine responses (Figure 7a), despite the complemented bacteria producing as much Tipα as WT bacteria (Figure S4) and being able to do so when added to THP‐1 cells as live organisms (Figure S11). Complementation also did not restore infectivity to *tipA* mutant bacteria in mice (Figure S3). It is possible that the packaging of Tipα in the EV membrane of the complemented bacteria differs to that in WT bacteria and/or that EV trafficking within epithelial cells is different from that in phagocytic cells.

Given that the isolation method can have profound effects on the characteristics of EV preparations (Takov et al. 2019), we co‐cultured THP‐1 derived macrophages with SEC EVs isolated from *H. pylori* WT, *tipA* mutant or complemented *tipA (tipA+)* bacteria. BHI broth that had been purified in the same manner as SEC EVs was used as a negative control. Surprisingly, in contrast to the findings for UC EVs, there were no differences in TNF or IL‐8 responses to the different SEC EVs (Figure 7d,e). Similar findings for SEC EVs were obtained in mouse WT and *Tlr2^−/−^Tlr4^−/−^* iMacs, whereas *tipA* EVs isolated by UC still induced greater amounts of TNF than WT EVs (Figure 7f). As expected, *Tlr2^−/−^Tlr4^−/−^* iMacs treated with SEC and UC EVs produced significantly less TNF than WT cells (Figure 7f). We suggest that the loss of some Tipα during the SEC procedure may have impacted the ability of Tipα to elicit immunomodulatory responses by those EVs. Overall, our studies have shown that Tipα has immunomodulatory roles but that this is impacted by the method of EV purification.

## 4 Discussion

Nucleomodulins are an emerging subset of virulence factors that localise to the host nucleus and disrupt host cell processes (Hanford et al. 2021). In previous work, it was reported that *H. pylori* Tipα is able to traffic to the nucleus (Suganuma et al. 2008), leading us to hypothesise that this protein may be a novel nucleomodulin. We addressed this hypothesis by studying Tipα in its native form rather than as a recombinant protein, thereby distinguishing this work from previous studies. Thus, we were able to confirm previous proteomics data showing that Tipα is present in *H. pylori* EVs (Turner et al. 2015) and, more significantly, demonstrate for the first time that most of the secreted Tipα in *H. pylori* culture supernatants is actually associated with EVs (Figure 2g,h) and encapsulated within the vesicles (Figure 4, Figure S6f–h). Furthermore, we show that EV‐associated Tipα traffics to the nuclear compartment and has immunomodulatory effects on host cells but this is dependent on the EV isolation method (Figure 7).

It was reported that *H. pylori* EVs are enriched in the gastric contents of gastric cancer patients (Choi et al. 2017). An enrichment of *H. pylori* EVs in the host would increase the bioavailability of EV‐associated Tipα, thus possibly contributing to disease progression. Intriguingly, isolates associated with cases of gastric cancer were reported to secrete greater amounts of Tipα (Suganuma et al. 2005; Suganuma et al. 2008). Although we observed varying levels of Tipα secretion between strains, we could not find a clear correlation with disease outcome (Figure 1b,c). Due to the limited number of clinical isolates analysed, however, it is not possible to reach a conclusion regarding correlations between Tipα secretion by *H. pylori* isolates and disease outcome. Thus, further investigation with a greater number of clinical isolates is warranted. Furthermore, whole cell preparations of the clinical isolates contained similar amounts of dimeric Tipα (Figure 1c), suggesting that the strain‐dependent differences observed may be attributed to Tipα secretion and not its synthesis. Given that most secreted Tipα is associated with EVs, it is instead possible that differences in vesiculation may explain the results. This would be consistent with the observation that EV production in *H. pylori* varies in a strain‐specific manner (Bitto et al. 2021). It would be interesting in future studies to determine if increased vesiculation correlates with worse disease outcomes.

Consistent with previous findings (Suganuma et al. 2008), we confirmed that rTipα can reach the nuclear compartment of human gastric epithelial cell lines (Figures 5 and 6). Moreover, we showed that Tipα in its native, EV‐associated form was also able to reach the nuclear compartment (Figure 6). Contrary to previous reports (Watanabe et al. 2010a), however, we were unable to show any evidence for nucleolin playing a role in Tipα internalisation and trafficking to the nucleus. We found nucleolin to be weakly expressed on the surface of a range of gastric epithelial cell lines and as expected, mainly concentrated to the nucleolus (Figure 6b–e). Also, EV‐associated Tipα was able to enter and undergo nuclear trafficking in AGS cells (Figure 5), which had only modest levels of nucleolin surface expression (Figure 6c,d). Finally, we were unable to demonstrate any evidence for binding of rTipα to nucleolin (Figure 6f,g). As reported previously (Kaparakis et al. 2010; Irving et al. 2014), *H. pylori* EVs are highly effective at entering non‐phagocytic cells and thus we suggest that EVs are sufficient to mediate Tipα entry into gastric epithelial cells and that surface nucleolin binding is dispensable (Figure S8).

Previous studies showed that rTipα had proinflammatory activity in cell lines (Suganuma et al. 2005; Wongsirisin et al. 2018; Morningstar‐Wright et al. 2022), a finding that was confirmed in the present work (Figure 7). It was also reported previously that WT *H. pylori* SS1 bacteria induced significantly higher expression levels of *Tnf* and other proinflammatory genes in the gastric mucosa of mice, when compared with mice infected with *tipA* mutant bacteria (Morningstar‐Wright et al. 2022). It was suggested that the reduced proinflammatory gene expression in the mice challenged with the *tipA* bacteria may be attributed to the reduced bacterial loads in those animals (Morningstar‐Wright et al. 2022). As shown in Figure S3, we also found that the colonisation ability of *H. pylori tipA* mutant bacteria was impaired, albeit more dramatically than reported previously for other *tipA* mutants (Godlewska et al. 2008; Morningstar‐Wright et al. 2022), and that gastric TNF production was also reduced in the Tipα ‐challenged mice. We suggest that Tipα may have an immunosuppressive role but only when associated with EVs purified by ultracentrifugation (Figure 7a–c) or with live bacteria (Figure S10). It seems that the form of Tipα may be an important determinant in its immunomodulatory properties, however, further research is required to clarify this question.

The use of SEC improved the uniformity of *H. pylori* EV preparations (Figure 3c–g), resulting also in purer preparations with less contaminating material, as observed when comparing the EM images of UC and SEC preparations (Figures 2f and 3h, respectively). The use of SEC, however, also resulted in a loss of some proteins from the preparations, including any Tipα that is not enclosed within the particles (Figure 3b, Figure S6b–e). The type of EV isolation method is known to alter EV characteristics and affect the outcome of even simple assays, like protein quantification (Bitto et al. 2017; Takov et al. 2019). UC and SEC have been shown to have different impacts on the molecules attached to the surface of EVs, known as the biomolecular corona, which then influences the physicochemical characteristics of EVs (Esmaeili et al. 2025). For example, UC was shown to strip and/or “soften” the associated corona (Wolf et al. 2022), whereas SEC was reported to remove many more proteins than centrifugation (Tóth et al. 2021). The UC method used in the current study utilised smaller‐pore filters for dialysis and concentration (i.e. 10 kDa MWCO), so it is likely that a greater amount of corona was retained than in the SEC method. Given that UC EVs seemed to have more Tipα per particle (Figure S6d), it is possible that EV‐associated Tipα may be associated with some form of corona. This might explain why the WT and *tipA (tipA+)* EVs isolated by SEC did not elicit greater cytokine responses than those of EVs isolated by SEC from *tipA* mutant bacteria. Very little is currently known regarding the corona in EVs of prokaryotic origin and thus further studies are needed, as well as on the association of Tipα with a possible corona in *H. pylori* EVs.

An interesting observation from the current work was that rTipα generated from *H. pylori* SS1 had lower immunogenicity when compared with the reference strain 26695 that was used in previous studies (Figure S9). Although *in silico* analyses indicated that Tipα was highly conserved in *H. pylori* strains and nearly all other gastric *Helicobacter* spp., *H. pylori* SS1 Tipα differed by the presence of a 20‐amino acid extension to the signal peptide sequence and two non‐synonymous amino acid substitutions when compared with that of 26695 (Figure S2). Nevertheless, as both recombinant proteins were produced without signal peptides, it is unlikely that the extended signal sequence had any impact on function and thus, was responsible for the differences in cytokine responses. It is also unclear whether the extended signal sequence is simply an annotation error. Regarding the non‐synonymous substitution at position 86 in SS1 Tipα, it is located at the beginning of the first alpha helix of a proposed dodecin domain (Tosi et al. 2009), so it is difficult to understand how this may impact its function. The second substitution is located between the third and fourth alpha helices that share similarity to protein structures with nucleic acid‐binding potential, for example Nab2 (Tosi et al. 2009). As Tipα has DNA binding activity (Figure 5) (Suganuma et al. 2005; Kuzuhara et al. 2007), it is possible that this substitution alters the DNA binding capacity of *H. pylori* SS1 Tipα, resulting in reduced immunogenicity in host cells.

In summary, we propose that *H. pylori* Tipα is a nucleomodulin that is predominantly secreted in association with EVs and not as a free soluble protein. Indeed, *H. pylori* Tipα was reported to be enriched in *H. pylori* EVs when compared with the bacteria (Zavan et al. 2019). Importantly, we now show that unlike several other well‐characterised EV‐associated proteins, Tipα is mainly encapsulated within the vesicles (Figure 4, Figure S6f–h). Given that Tipα appears to have been evolutionarily conserved amongst closely related gastric *Helicobacter* spp., together with the presented findings, we propose that it is likely to play an important biological role. Genomic analyses identified Tipα as being one of the virulence factors that has most diverged between East Asian and European *H. pylori* isolates (Kawai et al. 2011), however, the significance of this finding remains unclear. Despite previous reports, we did not find increased secretion of Tipα correlating with disease outcomes, though this requires further investigation. EV‐associated Tipα was shown to reach the nuclear compartment where it may then down‐modulate proinflammatory responses and facilitate bacterial colonisation. Consistent with this suggestion, mice that had been inoculated with *H. pylori* SS1 *tipA* mutant bacteria had significantly lower bacterial loads than those inoculated with WT bacteria (Figure S3) (Godlewska et al. 2008; Morningstar‐Wright et al. 2022). Our findings underline the importance of EVs as an underappreciated mechanism by which bacteria can secrete nucleomodulins, toxins and other effector molecules. It remains an open question as to why these factors are very often found to be associated with EVs when the bacteria have well‐defined secretion systems.

## Author Contributions

**Jack K. Emery**: methodology, investigation, formal analysis, writing – original draft, writing – review and editing, visualization, validation, funding acquisition. **Variya Nemidkanam**: methodology, investigation, formal analysis, visualization, validation, funding acquisition. **Nina Colon**: methodology, investigation, formal analysis, visualization, validation. **Kate R. J. Friesen**: methodology, investigation, formal analysis, resources, funding acquisition. Lena H. M. Le: investigation. **Angus R. Cramond**: investigation, formal analysis. **Maxine Yap**: investigation, formal analysis. **Georgie Wray‐McCann**: investigation. **Mônica S. Barbosa: investigation. David J. McGee**: resources, writing – review and editing. **Natalia Castaño‐Rodríguez**: resources, funding acquisition. **Dongmei Tong**: supervision. **Caroline Skene**: writing – review and editing, supervision. **Laurent Terradot**: resources, writing – review and editing. **Richard L. Ferrero**: conceptualization, methodology, formal analysis, supervision, funding acquisition, project administration, resources, writing – review and editing, visualization.

## Funding

This work was supported by funding to RLF from the Australian Research Council (Discovery grant DP210103881), the National Health and Medical Research Council (Ideas grants, APP2012620, APP2021710) and US Department of Defense (Peer Reviewed Cancer Research Program Idea Award no. CA200760, contract no. W81XWH2110953). JKE, LHML and AC were supported by scholarships from the Research Training Program (Australian Department of Education) and VN by a scholarship from the Royal Golden Jubilee Funding Scheme (Thailand). KF was the recipient of a Canadian Queen Elizabeth II Diamond Jubilee Scholarship and Mitacs Globalink Research Award (Canada). NCR was supported by the UNSW Scientia Fellowship program and a Cancer Australia/Pancare Foundation PdCCRS Early Career Researcher Grant (2012944). Research at the Hudson Institute of Medical Research is supported by the Victorian Government's Operational Infrastructure Support Program.

## Disclosure

The authors of this original research paper declare there are no competing interests.

## Conflicts of Interest

The authors report no conflict of interest.

## Geolocation Information

This work was completed at the Centre for Innate Immunity and Infectious Diseases, Hudson Institute of Medical Research and Department of Molecular and Translational Science, Monash University. 27–31 Wright Street, Clayton, Victoria, Australia, postal code 3168.

## Supporting information




**Figure S1**: EV isolation and detection of Tipα in bacterial culture supernatants.

**Figure S2**: Tipα is highly conserved amongst *H. pylori* isolates and closely related gastric Helicobacter spp.

**Figure S3**: Tipa‐deficient bacteria are unable to colonise mice.

**Figure S4**: Western blot analyses confirming the phenotypes of *H. pylori tipA* mutant and complemented *tipA* (*tipA*+) mutant bacteria.

**Figure S5**: TEM images of EVs isolated by ultracentrifugation.

**Figure S6**: Comparison of Tipα cargo loads in EVs prepared by SEC or UC.

**Figure S7**: *H. pylori* EVs harbouring Tipα localise to the perinuclear region of epithelial cells.

**Figure S8**: EVs enter host epithelial cells by endocytosis and macropinocytosis.

**Figure S9**: rTipα derived from *H. pylori* 26695 induces significantly stronger pro‐inflammatory responses than that from strain SS1.

**Figure S10**: Bacterial‐associated Tipα similarly modulates proinflammatory immune responses in THP‐1 cells.

**Table S1**: Description of plasmids and primers used in this study.

**Table S2**: Zetaview PMX‐130 sample, instrument and analysis parameters.

**Table S3**: Gastric and enterohepatic *Helicobacter* spp. used in *in silico* analyses.

Supplementary Videos

## Data Availability

All data related to the study are available from the corresponding author upon reasonable request.
