# Supplementary figures and images for: Outer Membrane Vesicles Mediate the Secretion and Nuclear Trafficking of a Bacterial Nucleomodulin
Source: J Extracell Vesicles. 2026 Apr 30;15(5):e70286. doi: 10.1002/jev2.70286 (PMC13132344; doi:10.1002/jev2.70286)

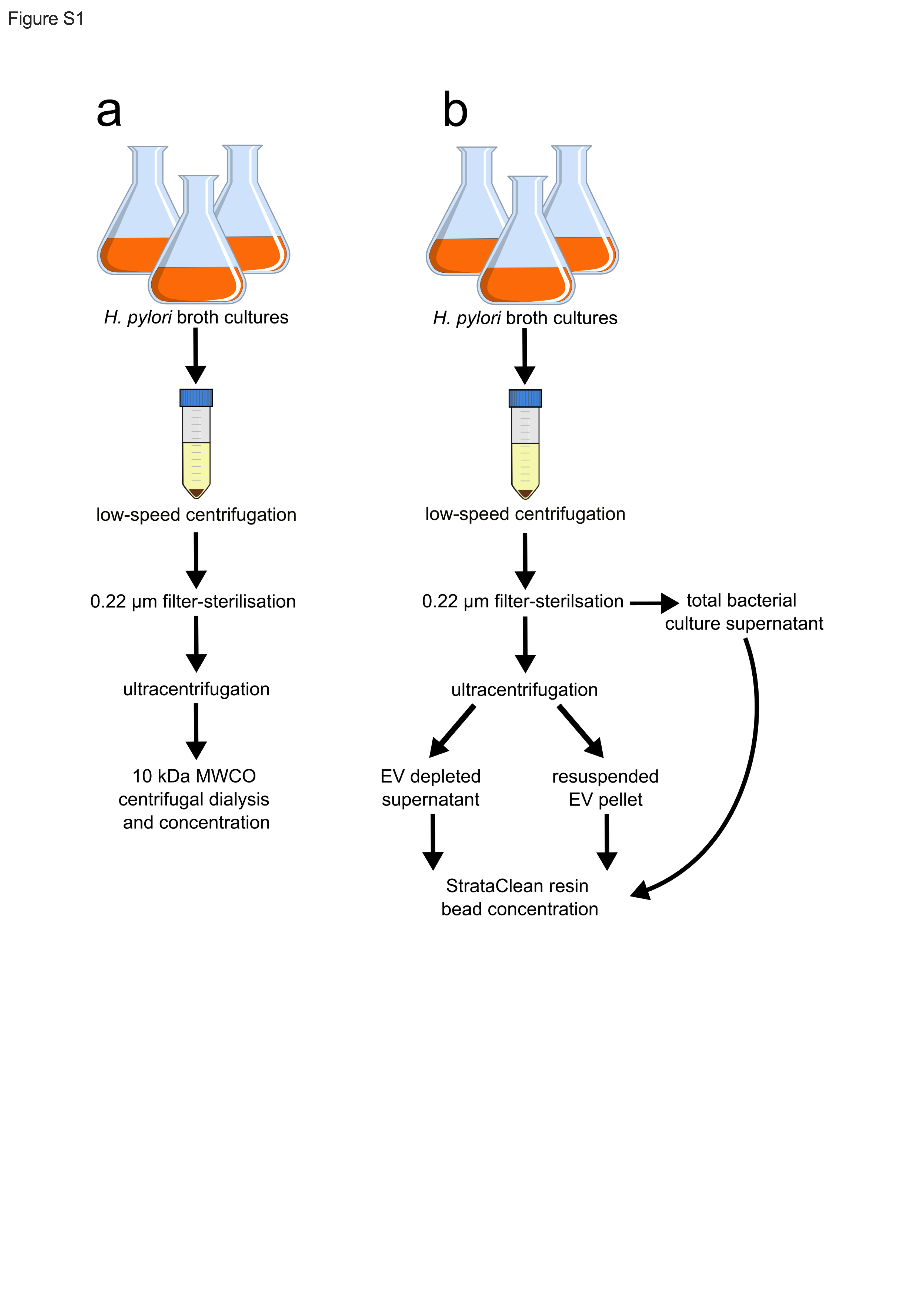

Supplement: Supplementary file 1 — Figure S1: EV isolation and detection of Tipα in bacterial culture supernatants. [file JEV2-15-e70286-s014.tiff]

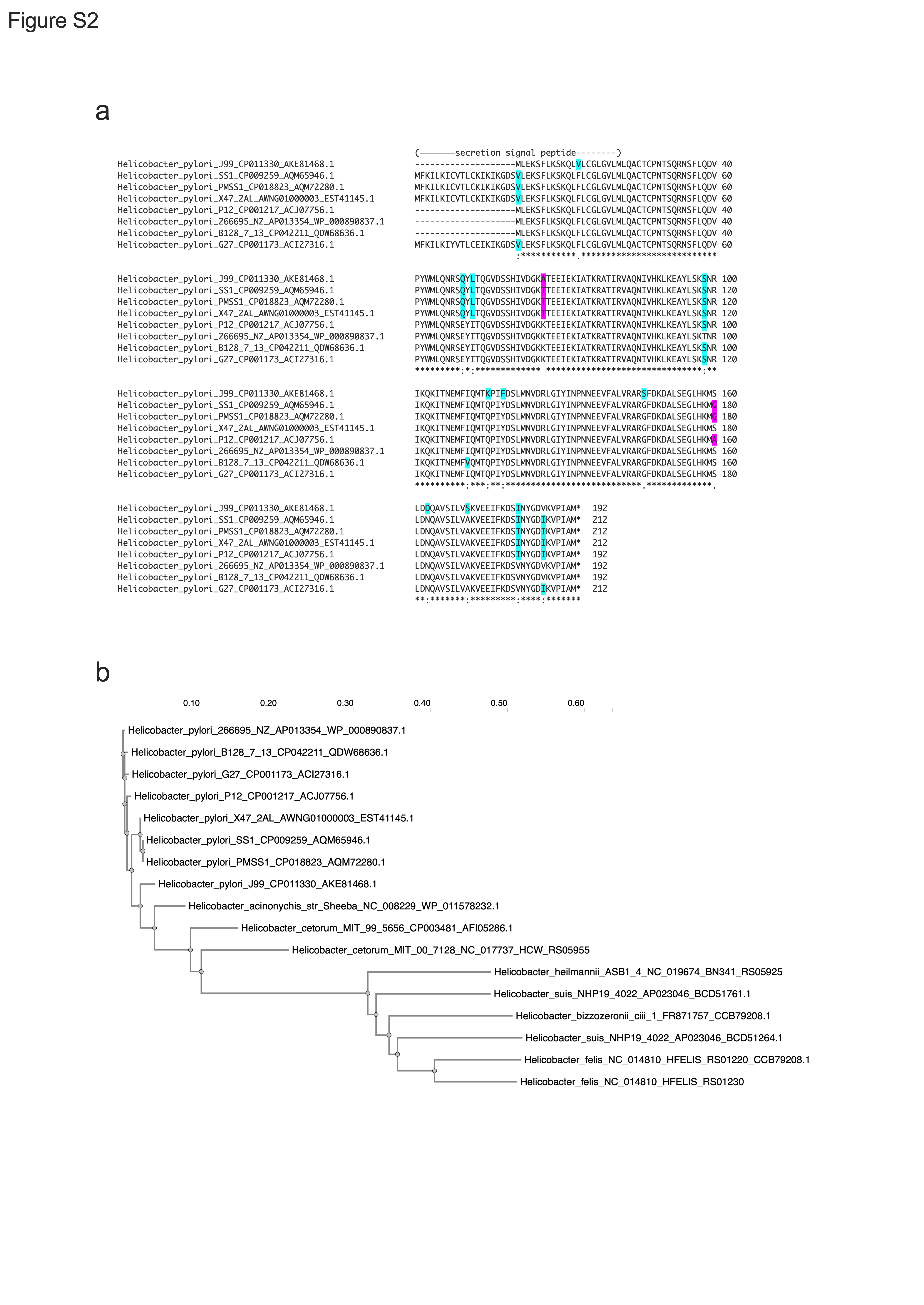

Supplement: Supplementary file 2 — Figure S2: Tipα is highly conserved amongst H. pylori isolates and closely related gastric Helicobacter spp. [file JEV2-15-e70286-s002.tiff]

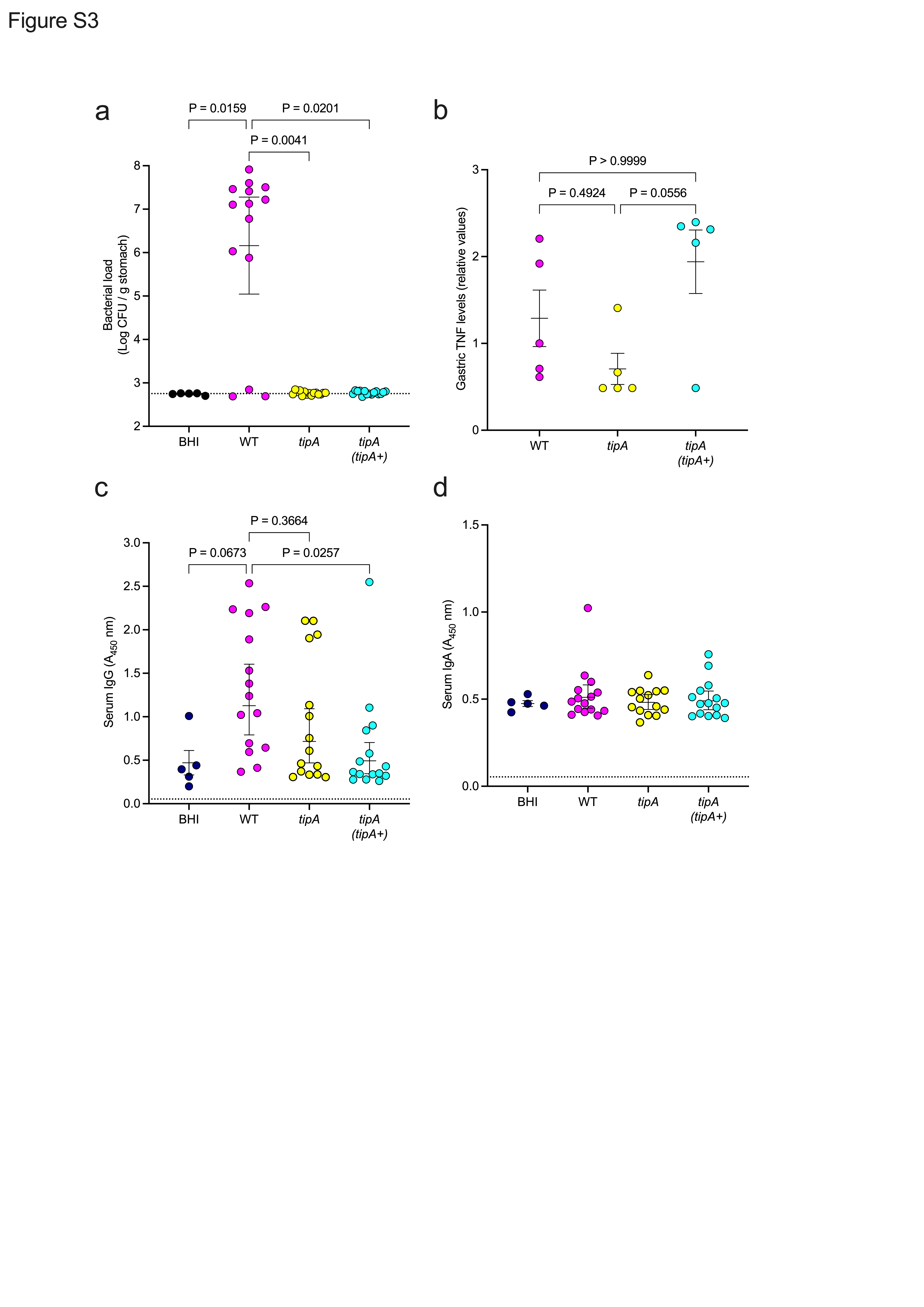

Supplement: Supplementary file 3 — Figure S3: Tipa‐deficient bacteria are unable to colonise mice. [file JEV2-15-e70286-s007.tiff]

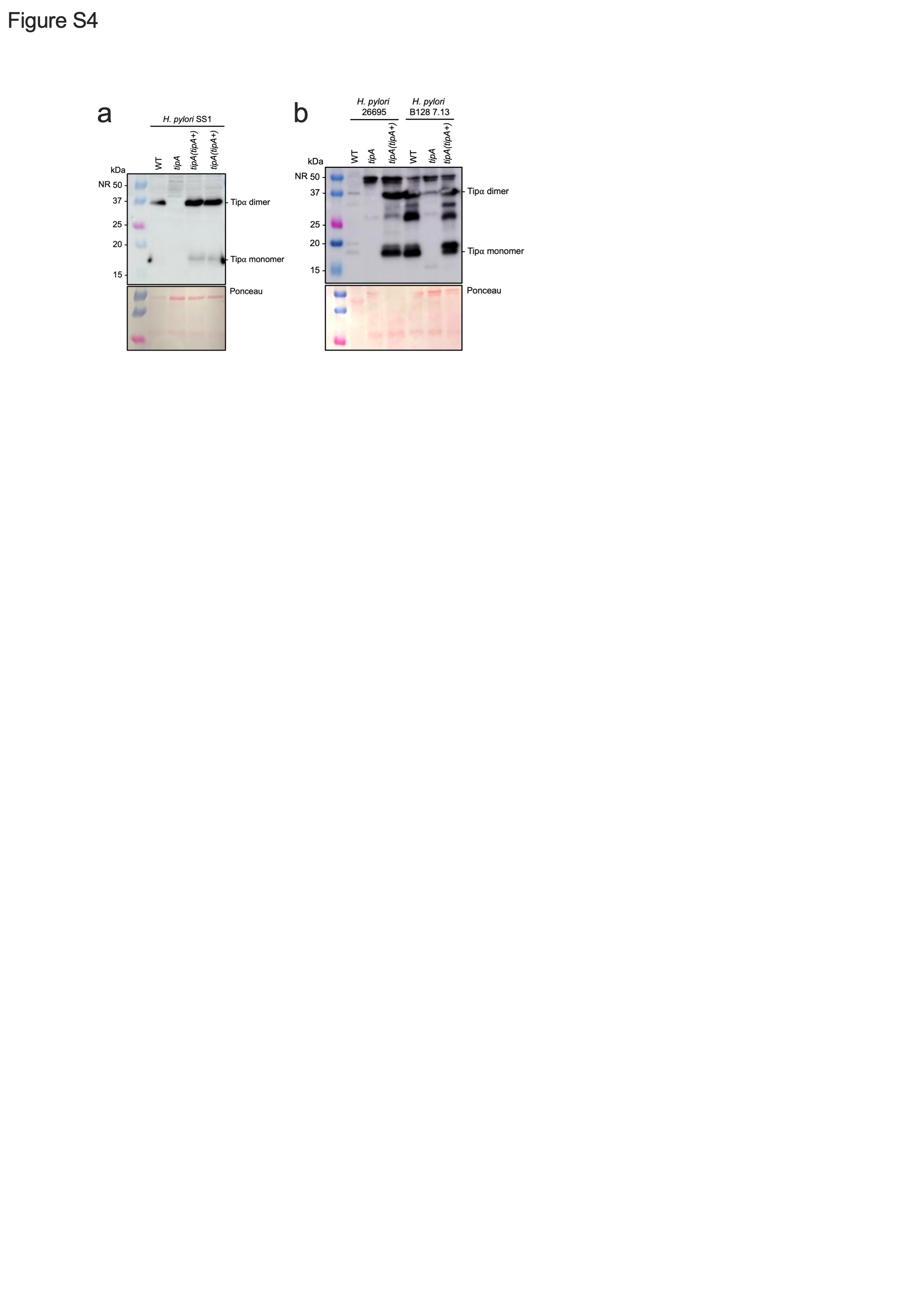

Supplement: Supplementary file 4 — Figure S4: Western blot analyses confirming the phenotypes of H. pylori tipA mutant and complemented tipA (tipA+) mutant bacteria. [file JEV2-15-e70286-s001.tiff]

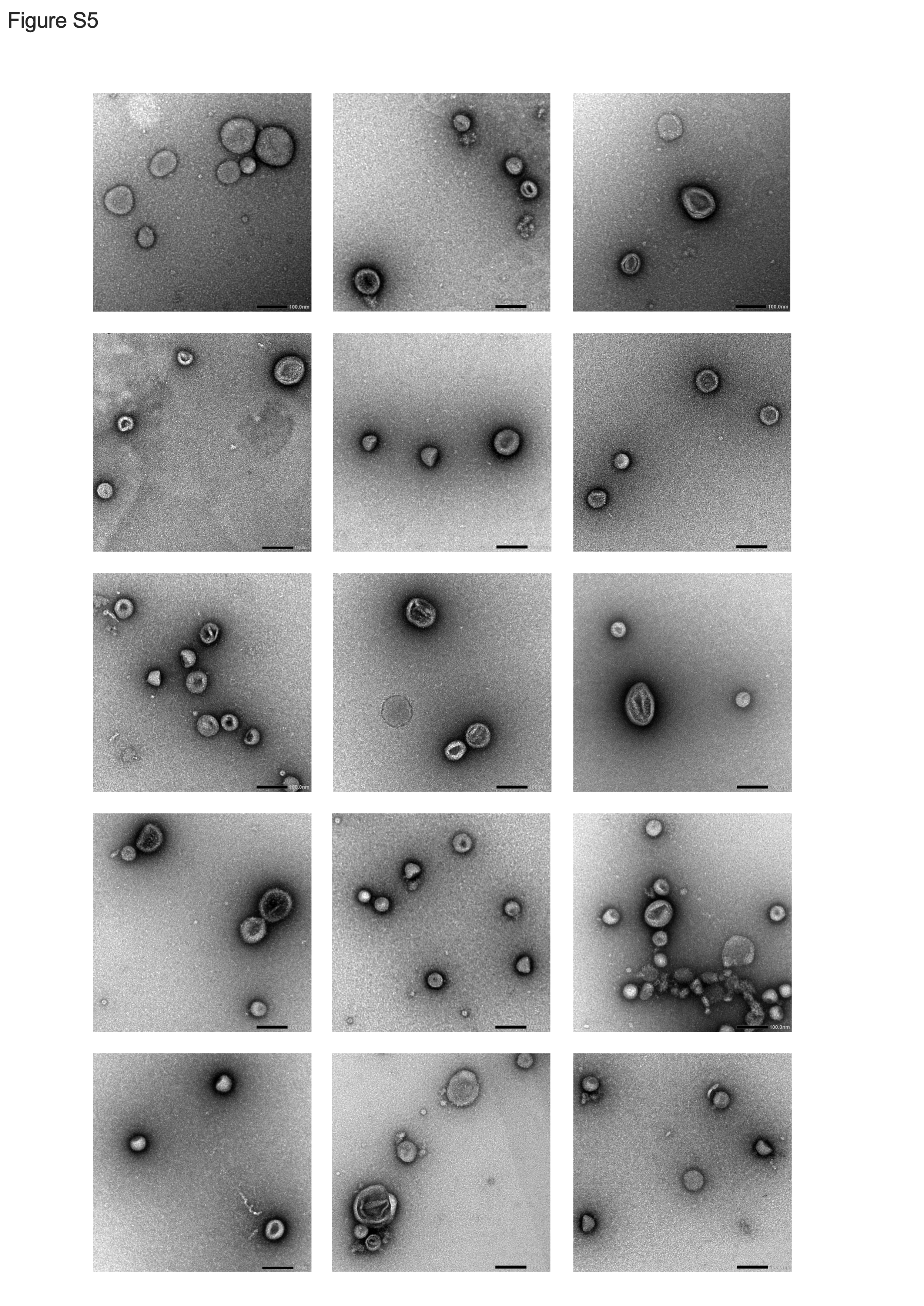

Supplement: Supplementary file 5 — Figure S5: TEM images of EVs isolated by ultracentrifugation. [file JEV2-15-e70286-s012.tiff]

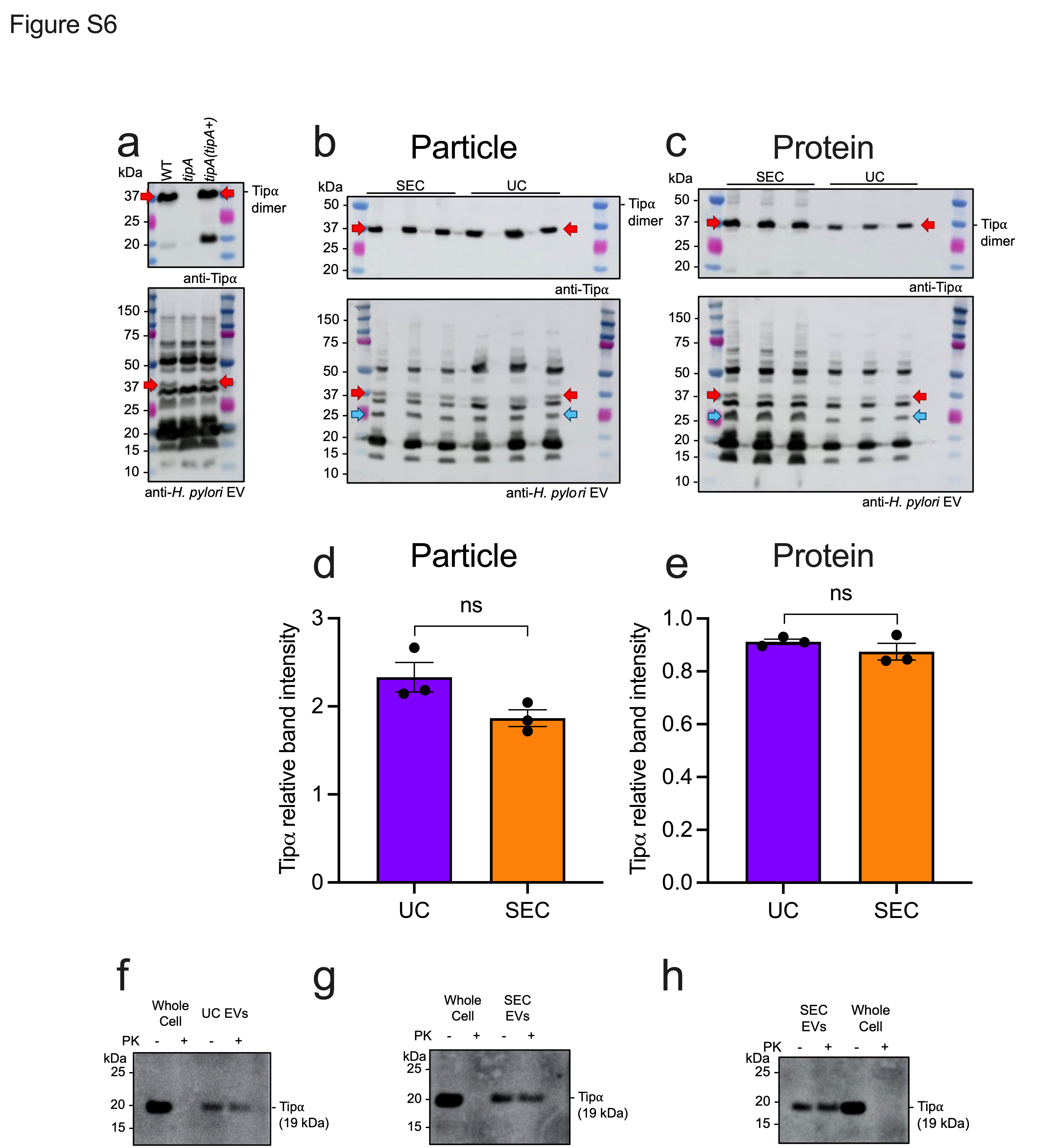

Supplement: Supplementary file 6 — Figure S6: Comparison of Tipα cargo loads in EVs prepared by SEC or UC. [file JEV2-15-e70286-s004.tiff]

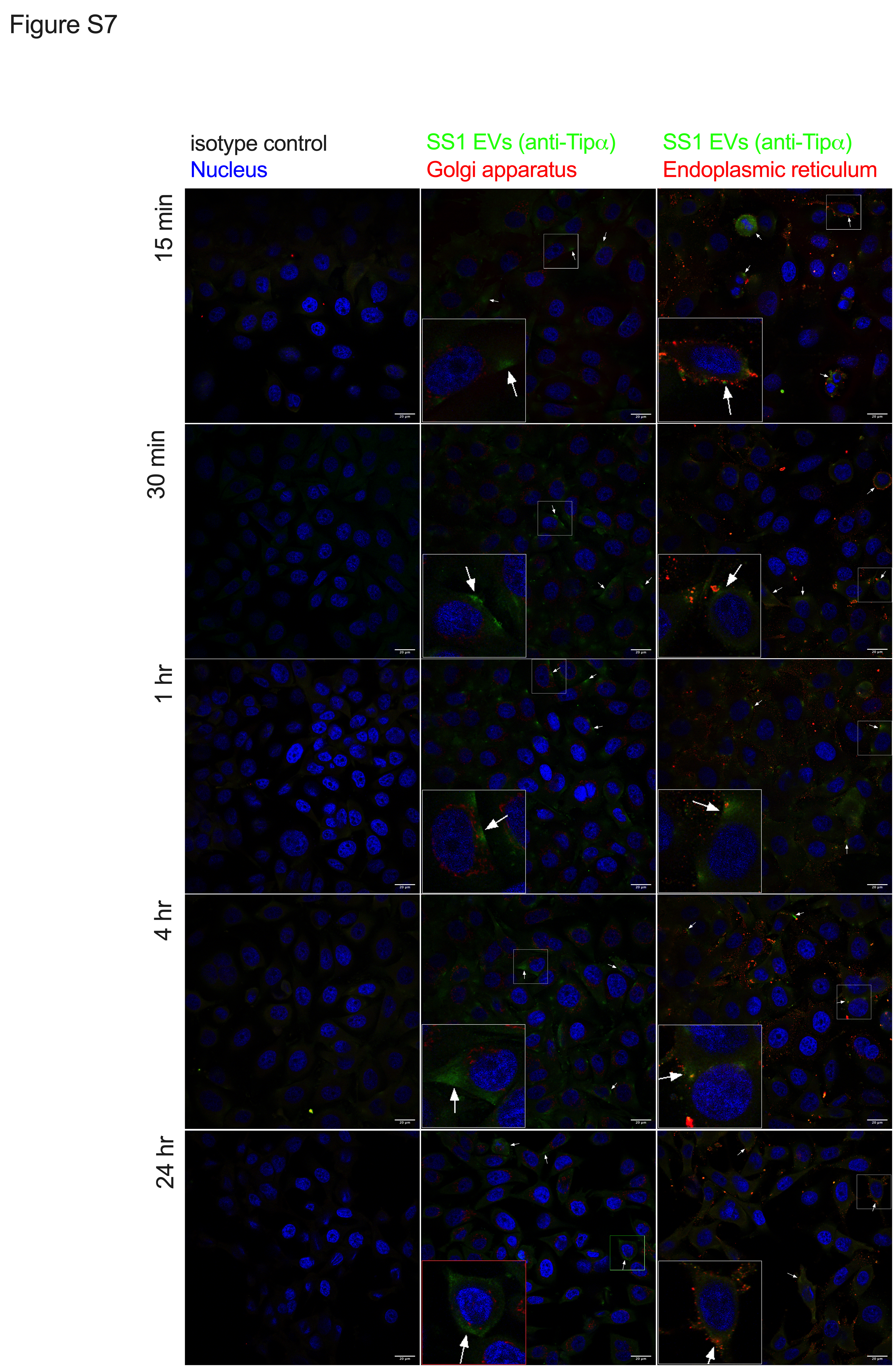

Supplement: Supplementary file 7 — Figure S7: H. pylori EVs harbouring Tipα localise to the perinuclear region of epithelial cells. [file JEV2-15-e70286-s006.tiff]

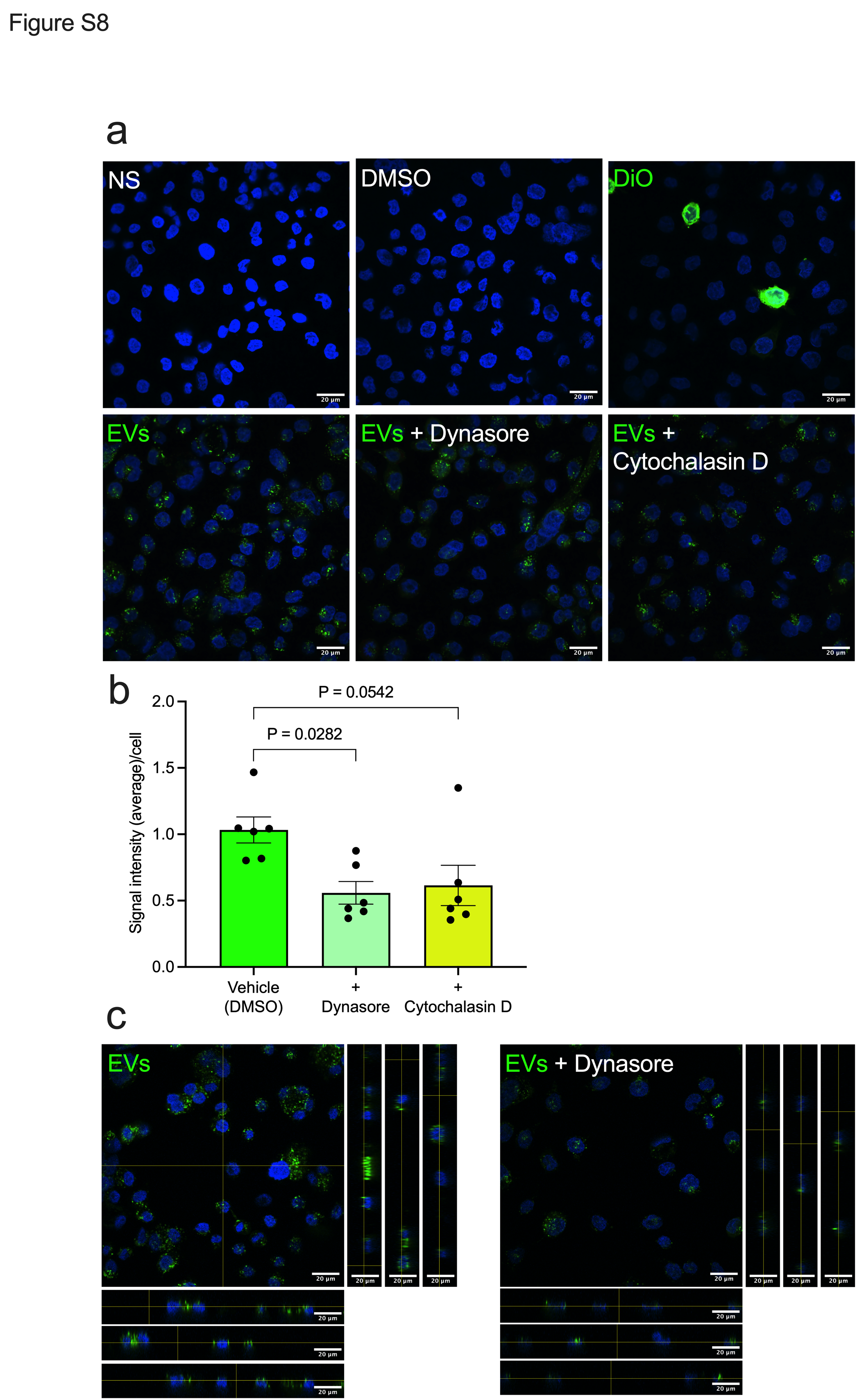

Supplement: Supplementary file 8 — Figure S8: EVs enter host epithelial cells by endocytosis and macropinocytosis. [file JEV2-15-e70286-s013.tiff]

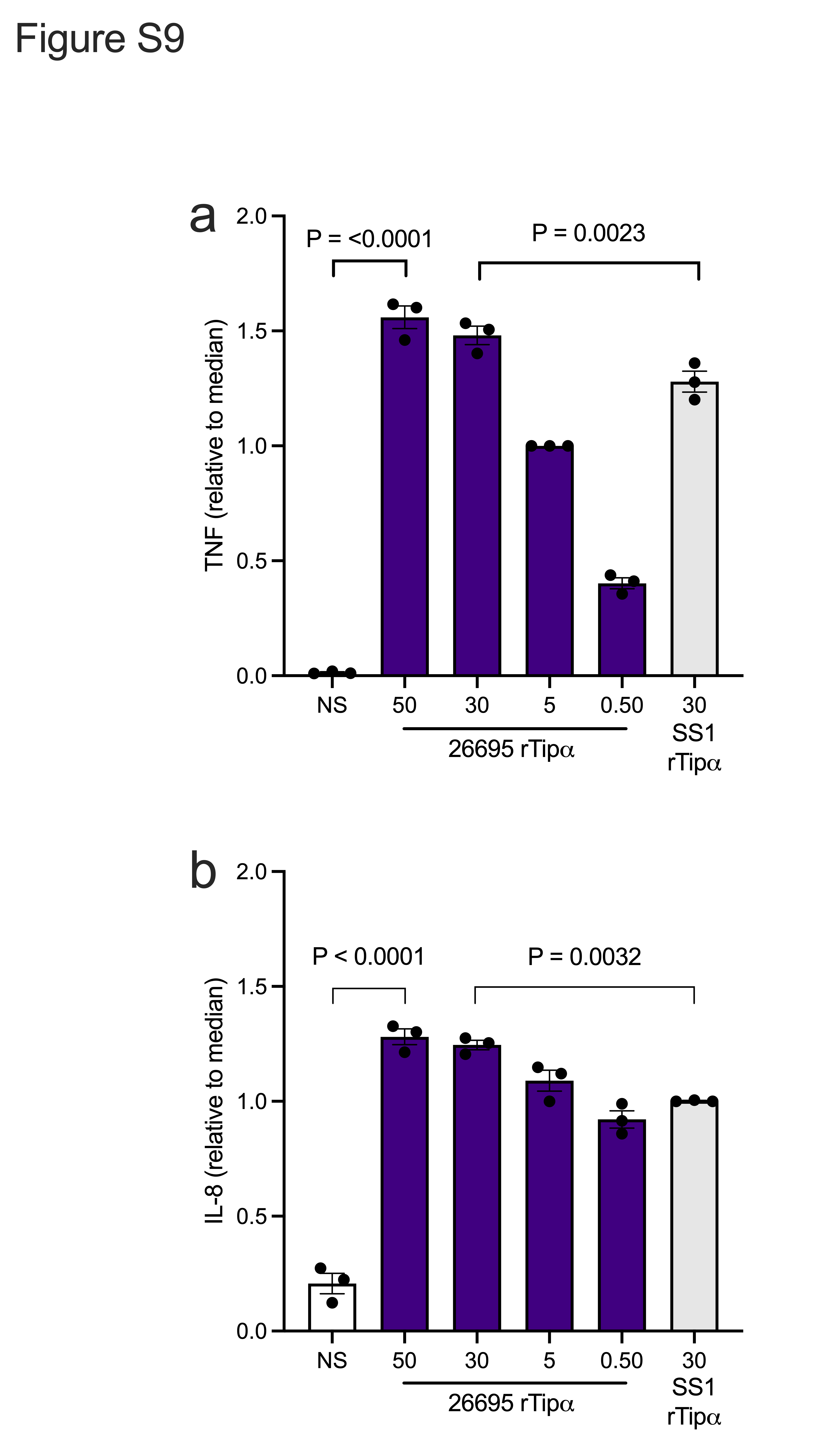

Supplement: Supplementary file 9 — Figure S9: rTipα derived from H. pylori 26695 induces significantly stronger pro‐inflammatory responses than that from strain SS1. [file JEV2-15-e70286-s008.tiff]

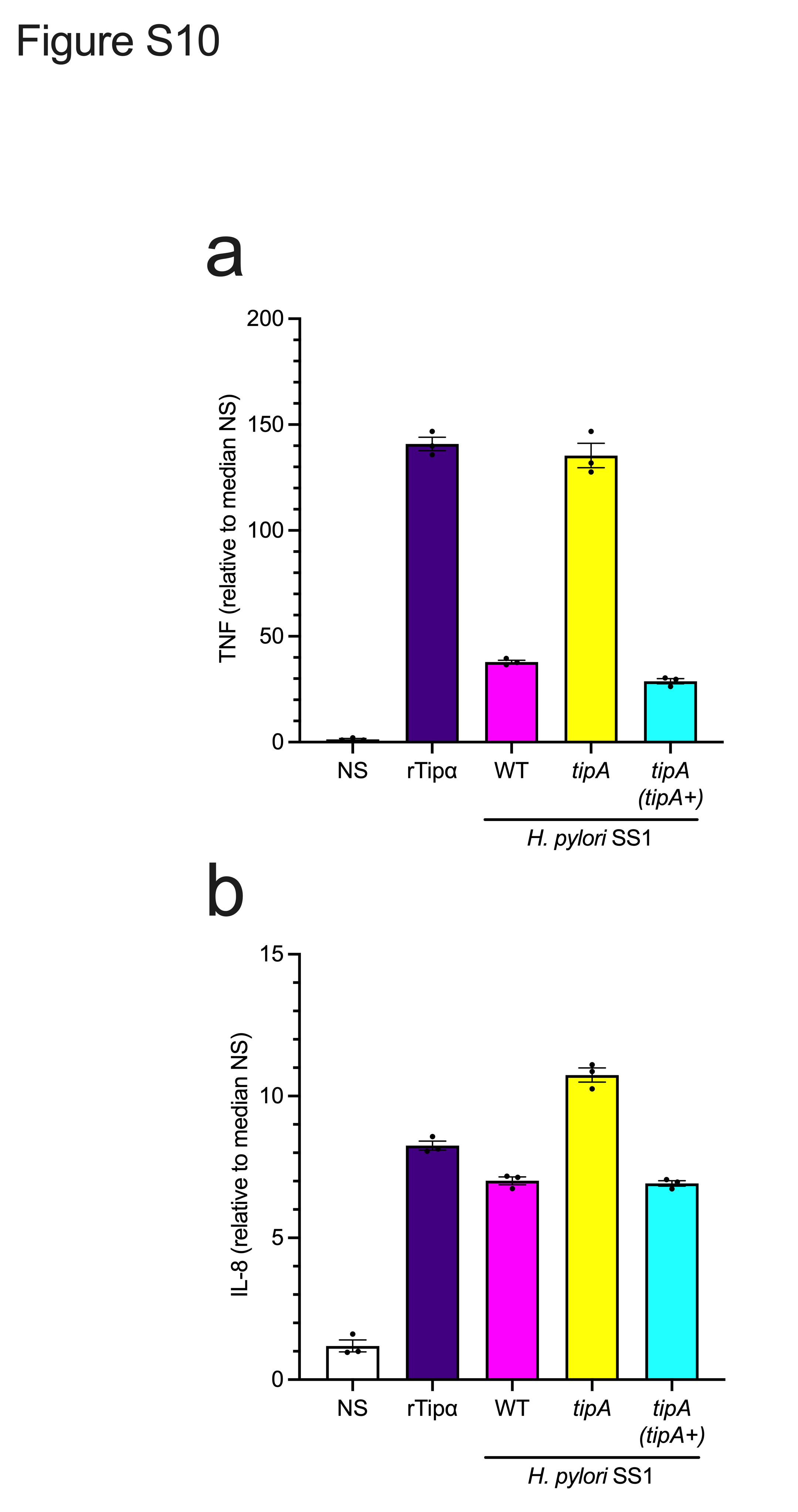

Supplement: Supplementary file 10 — Figure S10: Bacterial‐associated Tipα similarly modulates proinflammatory immune responses in THP‐1 cells. [file JEV2-15-e70286-s010.tiff]
