# Supplementary material for: Outer Membrane Vesicles Mediate the Secretion and Nuclear Trafficking of a Bacterial Nucleomodulin
Source: J Extracell Vesicles. 2026 Apr 30;15(5):e70286. doi: 10.1002/jev2.70286 (PMC13132344; doi:10.1002/jev2.70286)
Supplement: Supplementary file 12 — Table S2: Zetaview PMX‐130 sample, instrument and analysis parameters. [file JEV2-15-e70286-s003.docx]

**Supplementary figure 1**

**EV isolation and detection of Tipα in bacterial culture supernatants.** (a) Supernatants from overnight *H. pylori* cultures were harvested by low-speed centrifugation, then filter-sterilised to remove any remaining bacteria. Culture supernatants were ultracentrifuged to pellet EVs, before dialysis and final concentration using 10 kDa molecular weight cut-off filters. (b) Free, soluble and EV-associated Tipα were isolated from equivalent volumes of supernatant prior to ultracentrifugation, with EV-depleted supernatants containing only free soluble Tipα and the resuspended EV pellets containing only EV-associated Tipα. Samples were concentrated using StrataClean resin beads (Agilent Technologies, Mulgrave, Victoria, Australia).

**Supplementary figure 2**

**Tipα is highly conserved amongst *H. pylori* isolates and closely related gastric *Helicobacter*spp.** (a) Multiple sequence analysis of related *H. pylori* strains: J99, SS1, PMSS1, X47-2AL, P12, 26695, B128 7.13 and G27. Blue = conserved substitution, red = non-conserved substitution. (b) Phylogenetic tree presenting the relationship of Tipα amino acid sequences among *H. pylori* strains and other gastric *Helicobacter* spp. The tree was rooted from the sequence of *H. pylori* 26695 Tipα. Scale represents closeness of *H. pylori* strains and *Helicobacter* spp. based upon Tipα amino acid identity.

**Supplementary figure 3**

**Tipα-deficient bacteria are unable to colonise mice.**

C57/BL6 mice were inoculated via a single oral gavage with either *H. pylori* SS1 WT, *tipA* mutant or complemented *tipA(tipA+)* bacteria (approximately 1x10^8^ colony-forming units (CFU) administered/mouse). At 28 days post-infection, animals were euthanised and analysed for bacterial loads and TNF levels in the gastric mucosa. Sera were analysed for anti-*H. pylori* IgG and IgA antibodies. (a) Bacterial numbers are plotted as log CFU/gram of stomach tissue. The dotted line indicates the sensitivity of detection by culture. (b) TNF was detected in gastric homogenates of challenged animals. Each data point represents an animal and is normalised relative the median response across all animals. Anti-*H. pylori* (c) IgG and (d) IgA levels were detected in the serum of challenged animals by ELISA against *H. pylori* SS1 sonicate. The dotted lines indicate the level of assay sensitivity. Each data point represents an individual animal, with data pooled from three independent experiments, including the geometric means and 95% confidence intervals. Statistical significance was determined by one-way ANOVA test with multiple comparisons.

**Supplementary figure 4**

**Western blot analyses confirming the phenotypes of *H. pylori* *tipA* mutant and complemented *tipA*(*tipA+*) mutant bacteria.** Western blotting was performed on whole cell lysates of *H. pylori* WT, *tipA* mutant and complemented *tipA*(*tipA+*) bacteria generated on the following strains: (a) SS1, (b) 26695 or B128 7.13. Lysates were electrophoresed on non-reducing (NR) gels, then immunoblotted against anti-Tipα serum. Lysates from (a) two and (b) one independent clones of complemented *tipA*(*tipA+*) bacteria were analysed. Ponceau red stain was used to detect total proteins. Molecular weight markers are shown on the left of each panel. One independent experiment.

**Supplementary figure 5**

**TEM images of EVs isolated by ultracentrifugation.** Images confirm the purity of EV preparations and absence of non-EV material, including flagella. Images were taken at x 50,000 magnification, scale bars = 100 nm. Images are representative of five independent preparations.

**Supplementary figure 6**

**Comparison of Tipα cargo loads in EVs prepared by SEC or UC.** (a) Western blot analysis of *H. pylori* WT, *tipA* mutant and complemented *tipA(tipA+)* on SEC EVs using antisera to Tipα or *H. pylori* EVs. Western blotting analysis of *H. pylori* WT SEC and UC EVs loaded onto gels based on either (b, d) particle numbers (1x10^9^ particles loaded/sample) or (c, e) protein (2 μg loaded/sample). EVs were loaded in technical triplicates. Quantification of Tipα relative abundance in SEC and UC EV preparations based on either (d) particle or (e) protein loading. The relative amounts of Tipα (red arrows) in the preparations were determined relative to a reference protein (blue arrows) by densitometry, using ImageJ software (Fiji). Western blot detection of Tipα in bacterial whole cells, (f) UC EVs or (g, h) SEC EVs after digestion with Proteinase K. Representative of n=2 and n=3 independent preparations of UC and SEC EVs, respectively.

**Supplementary figure 7**

***H. pylori* EVs harbouring Tipα localise to the perinuclear region of epithelial cells.** MKN-1 cells were incubated with *H. pylori* SS1 EVs (50 μg/ml) for 15 minutes-24 hours. Cells were analysed as follows: EV-associated Tipα (green), Golgi apparatus (red, middle panels), endoplasmic reticulum (red, right panels) and nuclei (blue). Mouse IgG was used as an isotype control. Arrows indicate the localisation of EV-associated Tipα. Images: 60 X magnification, scale bar = 20μm. One independent experiment.

**Supplementary figure 8**

**EVs enter host epithelial cells by endocytosis and macropinocytosis.** (a) AGS gastric epithelial cells were pretreated with either DMSO vehicle, Dynasore, Cytochalasin D or left untreated (NS), prior to the addition of DiO-labelled SEC EVs or DiO alone, treated in the same manner as the EVs. Nuclei were labelled with Hoechst to enable cell enumeration. Extracellular fluorescence was quenched with trypan blue. (b) The average signal intensity per cell was measured per field of view and subtracted from the DiO alone control. Each data point refers to a field of view, pooled from two technical replicates plotted as the mean ± SEM. (c) Cells (>250 per treatment) were enumerated. Orthogonal views of AGS cells treated with DiO EVs or treated with Dynasore prior to the addition of DiO EVs. Scale bars = 20 μm. One independent experiment.

**Supplementary figure 9**

**rTipα derived from *H. pylori* 26695 induces significantly stronger pro-inflammatory responses than that from strain SS1.** THP-1 cells were incubated with varying amounts of either 26695 rTipα or SS1 rTipα, or left untreated (NS). (a) TNF and (b) IL-8 levels in culture supernatants were detected at 6- or 24-hours post-incubation, respectively. Each data point corresponds to the mean of three technical replicates from one independent experiment and normalised to the median value for each experiment. Data were combined from three independent experiments, with mean ± SEM values shown. Statistical analyses were performed by one-way ANOVA with Tukey’s multiple comparisons.

**Supplementary figure 10**

**Bacterial-associated Tipα similarly modulates proinflammatory immune responses in THP-1 cells.** (a) TNF and (b) IL-8 production in THP-1 cells for 6 and 24 hours with either rTipα (50 μg/ml), *H. pylori* SS1 WT, *tipA* or *tipA*/*tipA+* live bacteria. Bacteria were removed after 1-hour coincubation with cells. Each data point corresponds to the mean of three technical replicates from one independent experiment and normalised to the median value for the NS samples. Mean ± SEM values are shown.

**Supplementary Tables**

**Table S1** Description of plasmids and primers used in this study

| **Plasmid** | **Description** | **Source** |
| --- | --- | --- |
| pGEM-T Easy Vector | Cloning vector (ampicillin^R^; 3 kb) | Promega |
| pTipAKO | pGEM-T containing promoterless *H. pylori* 26695 *tipA* gene disrupted by insertion of a kanamycin resistance cassette (*aphA3*). | This study |
| pIR203C04 | pBluescript SK II SK(-) containing intergenic regions between *H. pylori* genes HP0203 and HP0204, with a chloramphenicol resistance cassette | (Langford *et al.*, 2006) |
| pIR203tipAC04 | pIR203C04 with *H. pylori* 26695 *tipA* gene and native promoter included | This study |
| pET-Tipa | pET151/D-topo with the *H. pylori* 26695 *tipA* gene lacking the secretion signal sequence (ampicillin^R^) | (Tosi *et al.*, 2009) |
| pET-28a-c(+) | Cloning vector (kanamycin^R^). | Novagen |
| pET-SS1-tipA | pET-28a-c(+) with the *H. pylori* SS1 *tipA* gene lacking its secretion signal sequence | This study |
|  |  |  |
| **Primers (5'-3')** | Lowercase letters denote restriction sites and extra nucleotides to facilitate cloning. |  |
| tipA Comp FWD2 BamHI | cgggatccTCTAATGCTCAACTGCGTG |  |
| tipA Comp REV2 | CAGACGATAGCGAGGAACGCTTGTTG |  |
| rtipA FWD BamHI | cgcggatccATGCTGCAGGCTTGCAC |  |
| rtipA REV SacI | cgagctcCTACATGGCTATAGGGAC |  |

**Table S2** Zetaview PMX-130 sample, instrument and analysis parameters

| **Sample parameters** | |
| --- | --- |
| Temperature | 20°C |
| pH | 7.0 |
| Conductivity | 40.00 *μ*S/cm |
| **Instrument parameters** | |
| Laser wavelength | 488 nm |
| Filter wavelength | Scatter |
| Sensitivity | 75.0 |
| Shutter | 100 |
| Frame rate | 30 frames per second |
| Video resolution | high |
| Size distribution | 1 cycle, 11 positions |
| Level of detection | 50-200 particles per frame |
| **Analysis Parameters** | |
| Software | Zetaview version 8.06.01 SP1 |
| Max area | 1000 nm |
| Min area | 10 nm |
| Min brightness | 30 |
| nm/Class | 5 |
| Trace length | 30 |

**Table S3** Gastric and enterohepatic *Helicobacter* spp. used in *in silico* analyses.

| **Gastric**  ***Helicobacter* spp.** | **Strain** | **Genome accession no.** | ***tipA* gene accession** | **Tipα protein_ID** | **Identity(%)*** |
| --- | --- | --- | --- | --- | --- |
| *Helicobacter pylori* | J99 | CP011330 | YH61_02070 | AKE81468.1 | 94.27 |
| *Helicobacter pylori* | SS1 | CP009259 | HPYLSS1_00801 | AQM65946.1 | 95.83 |
| *Helicobacter pylori* | PMSS1 | CP018823 | HPYLPMSS1_00801 | AQM72280.1 | 95.83 |
| *Helicobacter pylori* | X47-2AL | AWNG01000003 | N871_00505 | EST41145.1 | 96.35 |
| *Helicobacter pylori* | P12 | CP001217 | HPP12_0603 | ACJ07756.1 | 97.92 |
| *Helicobacter pylori* | 26695 | NZ_AP013354 | HP_RS02940 | WP_000890837.1 | 100% |
| *Helicobacter pylori* | B128 7.13 | CP042211 | D5R83_03810 | QDW68636.1 | 98.96 |
| *Helicobacter pylori* | G27 | CP001173 | HPG27_556 | ACI27316.1 | 98.44 |
| *Helicobacter acinonychis* | Sheeba | NC_008229 | HAC_RS06010 | WP_011578232.1 | 89.58 |
| *Helicobacter bizzozeronii* | CIII-1 | FR871757 | HBZC1_02220 | CCB79208.1 | 39.04 |
| *Helicobacter cetorum* | MIT 00-7128 | NC_017737 | HCW_RS05955 | HCW_RS05955 | 73.68 |
| *Helicobacter cetorum* | MIT 99-5656 | CP003481 | HCD_01270 | AFI05286.1 | 82.29 |
| *Helicobacter felis* | - | NC_014810 | HFELIS_RS01220 | WP_013468718.1 | 36.46 |
| *Helicobacter felis* | - | NC_014810 | HFELIS_RS01230 |  | 36.96 |
| *Helicobacter pullorum* | NCTC13154 | LR134509 | - | - | - |
| *Helicobacter suis* | NHP19-4022 | AP023046 | NHP194022_09350 | BCD51264.1 | 36.31 |
| *Helicobacter suis* | NHP19-4022 | AP023046 | NHP194022_14320 | BCD51761.1 | 41.58 |
| **Enterohepatic *Helicobacter* spp.** | **Strain** | **Genome accession** | ***tipA* gene accession** | **Tipα protein_ID** | **Identity(%)*** |
| *Helicobacter bilis* | WiWa | GCF_000364285.1 | N.A. | N.A. | N.A. |
| *Helicobacter canadensis* | MIT 98-549 | CM000776 | N.A. | N.A. | N.A. |
| *Helicobacter canis* | NCTC 12740 | KI669458 | N.A. | N.A. | N.A. |
| *Helicobacter cinaedi* | PAGU611 | NC_017761 | N.A. | N.A. | N.A. |
| *Helicobacter fennelliae* | NCTC11613 | UGIB01000001, UGIB01000002 | N.A. | N.A. | N.A. |
| *Helicobacter hepaticus* | ATCC 51449 | NC_004917 | N.A. | N.A. | N.A. |
| *Helicobacter macacae* | MIT 99-5501 | GCF_000507845.1 | N.A. | N.A. | N.A. |
| *Helicobacter pametensis* | ATCC 51478 | GCF_000518225.1 | N.A. | N.A. | N.A. |
| *Helicobacter rodentium* | ATCC 700285 | GCF_000687535.1 | N.A. | N.A. | N.A. |
| *Helicobacter winghamensis* | ATCC BAA-430 | CP063533 | N.A. | N.A. | N.A. |

***** Amino acid identities were determined by alignment against Tipα from *H. pylori* 26695, using the Clustal Omega program.

N.A. Not applicable
