# Supplementary material for: Outer Membrane Vesicles Mediate the Secretion and Nuclear Trafficking of a Bacterial Nucleomodulin
Source: J Extracell Vesicles. 2026 Apr 30;15(5):e70286. doi: 10.1002/jev2.70286 (PMC13132344; doi:10.1002/jev2.70286)
Supplement: Supplementary file 13 — Table S3: Gastric and enterohepatic Helicobacter spp. used in in silico analyses. [file JEV2-15-e70286-s009.docx]

**Table S3** Gastric *Helicobacter* spp. used in *in silico* analyses.

| Gastric  *Helicobacter* spp. | Strain | Genome accession no. | *tipA* gene accession | Tipα protein_ID | Identity (%)* |
| --- | --- | --- | --- | --- | --- |
| *Helicobacter pylori* | J99 | CP011330 | YH61_02070 | AKE81468.1 | 94.27 |
| *Helicobacter pylori* | SS1 | CP009259 | HPYLSS1_00801 | AQM65946.1 | 95.83 |
| *Helicobacter pylori* | PMSS1 | CP018823 | HPYLPMSS1_00801 | AQM72280.1 | 95.83 |
| *Helicobacter pylori* | X47-2AL | AWNG01000003 | N871_00505 | EST41145.1 | 96.35 |
| *Helicobacter pylori* | P12 | CP001217 | HPP12_0603 | ACJ07756.1 | 97.92 |
| *Helicobacter pylori* | 26695 | NZ_AP013354 | HP_RS02940 | WP_000890837.1 | 100% |
| *Helicobacter pylori* | B128 7.13 | CP042211 | D5R83_03810 | QDW68636.1 | 98.96 |
| *Helicobacter pylori* | G27 | CP001173 | HPG27_556 | ACI27316.1 | 98.44 |
| *Helicobacter acinonychis* | Sheeba | NC_008229 | HAC_RS06010 | WP_011578232.1 | 89.58 |
| *Helicobacter bizzozeronii* | CIII-1 | FR871757 | HBZC1_02220 | CCB79208.1 | 39.04 |
| *Helicobacter cetorum* | MIT 00-7128 | NC_017737 | HCW_RS05955 | HCW_RS05955 | 73.68 |
| *Helicobacter cetorum* | MIT 99-5656 | CP003481 | HCD_01270 | AFI05286.1 | 82.29 |
| *Helicobacter felis* | N.A. | NC_014810 | HFELIS_RS01220 | WP_013468718.1 | 36.46 |
| *Helicobacter felis* | N.A. | NC_014810 | HFELIS_RS01230 | WP_231844187.1 | 36.96 |
| *Helicobacter mustelae* | NCTC12198 | LS483446 | N.D. | N.D. | N.D. |
| *Helicobacter pullorum* | NCTC13154 | LR134509 | N.D. | N.D. | N.D. |
| *Helicobacter suis* | NHP19-4022 | AP023046 | NHP194022_09350 | BCD51264.1 | 36.31 |
| *Helicobacter suis* | NHP19-4022 | AP023046 | NHP194022_14320 | BCD51761.1 | 41.58 |

***** Amino acid identities were determined by alignment against Tipα from *H. pylori* 26695, using the Clustal Omega program.

N.A. = not applicable. N.D. not detected.

**Table S3 (continued)** Enterohepatic *Helicobacter* spp. used in *in silico* analyses.

| Enterohepatic  *Helicobacter* spp. | Strain | Genome accession | *tipA* gene accession | Tipα protein_ID | Identity (%)* |
| --- | --- | --- | --- | --- | --- |
| *Helicobacter bilis* | WiWa | GCF_000364285.1 | N.D. | N.D. | N.D. |
| *Helicobacter canadensis* | MIT 98-549 | CM000776 | N.D. | N.D. | N.D. |
| *Helicobacter canis* | NCTC 12740 | KI669458 | N.D. | N.D. | N.D. |
| *Helicobacter cinaedi* | PAGU611 | NC_017761 | N.D. | N.D. | N.D. |
| *Helicobacter fennelliae* | NCTC11613 | UGIB01000001,  UGIB01000002 | N.D. | N.D. | N.D. |
| *Helicobacter hepaticus* | ATCC 51449 | NC_004917 | N.D. | N.D. | N.D. |
| *Helicobacter macacae* | MIT 99-5501 | GCF_000507845.1 | N.D. | N.D. | N.D. |
| *Helicobacter pametensis* | ATCC 51478 | GCF_000518225.1 | N.D. | N.D. | N.D. |
| *Helicobacter rodentium* | ATCC 700285 | GCF_000687535.1 | N.D. | N.D. | N.D. |
| *Helicobacter winghamensis* | ATCC BAA-430 | CP063533 | N.D. | N.D. | N.D. |

***** Amino acid identities were determined by alignment against Tipα from *H. pylori* 26695, using the Clustal Omega program.

N.A. = not applicable. N.D. = not detected.
